# Whole-genome sequencing reveals rare variants associated with gout in Taiwanese males

**DOI:** 10.3389/fgene.2024.1423714

**Published:** 2024-09-25

**Authors:** Yu-Ping Tseng, Ya-Sian Chang, Venugopala R. Mekala, Ting-Yuan Liu, Jan-Gowth Chang, Grace S. Shieh

**Affiliations:** ^1^ Institute of Statistical Science, Academia Sinica, Taipei, Taiwan; ^2^ Department of Pathology, Chung Shan Medical University Hospital, Taichung, Taiwan; ^3^ Department of Medical Research, China Medical University Hospital, Taichung, Taiwan; ^4^ Department of Laboratory Medicine, China Medical University Hospital, Taichung, Taiwan; ^5^ Bioinformatics Program, Taiwan International Graduate Program, Academia Sinica, Taipei, Taiwan; ^6^ Data Science Degree Program, Academia Sinica and National Taiwan University, Taipei, Taiwan; ^7^ Genome and Systems Biology Degree Program, Academia Sinica and National Taiwan University, Taipei, Taiwan

**Keywords:** association study, CADD, gout, rare variant, serum urate, whole-genome sequencing

## Abstract

To identify rare variants (RVs) of gout, we sequenced the whole genomes of 321 male gout patients and combined these with those of 64 male gout patients and 682 normal controls at Taiwan Biobank. We performed ACAT-O to identify 682 significant RVs (*p* < 3.8 × 10^−8^) clustered on chromosomes 1, 7, 10, 16, and 18. To prioritize causal variants effectively, we sifted them by Combined Annotation-Dependent Depletion score >10 or |effect size| ≥ 1.5 for those without CADD scores. In particular, to the best of our knowledge, we identified the rare variants rs559954634, rs186763678, and 13-85340782-G-A for the first time to be associated with gout in Taiwanese males. Importantly, the RV rs559954634 positively affects gout, and its neighboring gene *NPHS2* is involved in serum urate and expressed in kidney tissues. The kidneys play a major role in regulating uric acid levels. This suggests that rs559954634 may be involved in gout. Furthermore, rs186763678 is in the intron of *NFIA* that interacts with *SLC2A9,* which has the most significant effect on serum urate. Note that gene-gene interaction *NFIA-SLC2A9* is significantly associated with serum urate in the Italian MICROS population and a Croatian population. Moreover, 13-85340782-G-A significantly affects gout susceptibility (odds ratio 6.0; *P* = 0.038). The >1% carrier frequencies of these potentially pathogenic (protective) RVs in cases (controls) suggest the revealed associations may be true; these RVs deserve further studies for the mechanism. Finally, multivariate logistic regression analysis shows that the rare variants rs559954634 and 13-85340782-G-A jointly are significantly associated with gout susceptibility.

## Introduction

Gout is a joint and excruciating inflammatory arthritis caused by hyperuricemia and inflammation dysregulation. Furthermore, gout and serum urate levels are heritable. Several genes reported to be associated with serum uric acid (SUA) or gout are involved in the renal urate transporter system, such as *SLC22A12* (Enomoto et al., 2002), *SLC2A9* (Matsuo et al., 2008; Dinour et al., 2010) and *ABCG2* (Matsuo et al., 2009; Higashino et al., 2017) that modulate uric acid levels (Reginato et al., 2012; Chen et al., 2018). However, the pathogenic mechanisms of hyperuricemia and gout differ. Furthermore, although many common variants of gout have been discovered, they can not fully explain its susceptibility. Moreover, most of the 400 million detected variants from the ∼53 K diverse human genomes are rare, and functional variants tend to be rare (Taliun et al., 2021). Thus, we aim to discover novel rare variants that may cause gout in this study.

Many common variants associated with gout have been reported, e.g., rs22331142 in *ABCG2* (Chen et al., 2018) in a Taiwanese population. Matsuo and colleagues sequenced *ABCG2* to reveal multiple common and rare variants in a Japanese population (Higashino et al., 2017). Nevertheless, most of the variants for gout identified thus far are common variants (MAF > 5%). In this study, we aim to identify rare variants (MAF < 1%) that are associated with gout in male Taiwanese, as rare variants can have much larger per-allele effect sizes than common variants (Cheng et al., 2022). We integrated whole genome sequencing (WGS) data of 321 male gout patients from China Medical University Hospital (CMUH), 64 male gout patients, and 682 normal controls from Taiwan Biobank (TWB), to reveal rare variants associated with gout disease.

As rare variants appear very infrequently in the population, set-based methods that jointly analyze variants in a set are much more powerful than the single-variant analysis applied to common variants in genome-wide association studies (GWASs). Thus, we applied ACAT-O (Liu et al., 2019), which combines the strength of the sequence kernel association test (SKAT; Wu et al., 2011), the burden test (Li and Leal, 2008; Madsen and Browning, 2009; Price et al., 2010) and ACAT-V test (Liu et al., 2019). The power of ACAT-O to detect rare variants is robust against the sparsity of causal variants and the directionality of effects. After the rare variants associated with gout are detected, we further sift these variants by their Combined Annotation-Dependent Depletion (CADD (Kircher et al., 2014; Rentzsch et al., 2019; van der Velde et al., 2015), scores. CADD is a widely used measure of variant deleteriousness, and it can prioritize causal variants in genetic analysis. Next, we compute the odds ratio and proportion test of the rare variants with CADD ≥10 or |effect size| ≥ 1.5 for those without CADD scores.

## Materials and methods

### Study population

This study aimed to identify genes and rare variants associated with gout in Taiwanese male patients. Because there were no normal controls at China Medical University Hospital(CMUH henceforth, Taichung, Taiwan), we integrated the WGS data from CMUH with those from the Taiwan Biobank (TWB henceforth, Taipei, Taiwan) database. There were 321 male gout patients recruited at CMUH; 64 male gout patients and 682 normal controls (with serum urate level <6 mg/dL; according to the 2012 guideline of the American College of Rheumatology) from TWB, which provided WGS data, age, gout annotation, and serum urate levels for each sample. The 746 WGS data from TWB were the total male subjects sequenced when we started this investigation. The clinical information of gout patients and normal control analyzed are in Supplementary Table S1.

TWB is a database established in 2012 for research, a prospective study of 200,000 individuals aged 30–70 recruited from Taiwan. TWB consists of individuals, predominantly of Han Chinese ancestry, whose genomic profiles were integrated with lifestyle patterns to study the relationships between diseases and genetics. All participants underwent biochemical tests with blood and urine specimens and physical examination; they all signed informed consent for genotyping. The phenotype of gout disease was the self-report of physician-diagnosed gout, which was reported to retain good reliability and sensitivity (McAdams et al., 2011).

The IRB-BM committee of Academia Sinica (AS-IRB02-113170) and the CMUH Institutional Review Board in Taiwan (CMUH108-REC1-091) approved this study. All data from human participants were obtained from CMUH and the Taiwan Biobank database, for which data sharing and data linkage were parts of the consent, so a waiver of consent was granted by the CMUH IRB. Both the Declaration of Helsinki and the Good Clinical Practice Guidelines were followed, and informed consent was signed by all participants.

### Genotyping

The whole genomes of male gout patients at CMUH and participants of TWB were sequenced by the Illumina NovaSeq 6000 platform at least 30x depth, mapped to reference genome (hg38) by Dragon and BWA, and variant calling (.gvcf) performed by Dragon and GATK, respectively; both .gvcf files were annotated by VEP (McLaren et al., 2016). As we only assessed .vcf files from TWB, we combined the .bed files (converted by PLINK version 1.9) from the two sources. Because the batch effect of the union of the called variants from both sites could not be adjusted well even using 99 principle components (PCs), we intersected both sets of variants to result in the overlappings, and we called these SNPs CMUH_TWB data henceforth.

### Quality control

The standard per-individual and per-marker quality control (Anderson, et al., 2010) were performed on the CMUH_TWB data using PLINK 1.9 software. We first adopted the procedure of per-individual quality control. Individuals were excluded if identification of 1) individuals with incorrectly recorded or missing sex status, 2) individuals with genotyping call rates below 90% or outlying heterozygosity rate, namely homozygosity rate out of the sample mean ±3 s.e. confidence bounds, 3) individuals of divergent ancestry, or 4) uric acid levels missing. Moreover, we implemented per-marker quality control procedures to remove SNPs if 1) genotyping call rate below 100% (i.e., no missing allowed) or significant deviation from Hardy-Weinberg equilibrium in the controls (*p* < 0.001 and MAF >1%).

Finally, SNPs with MAF <1% were called to result in 8,703,559 rare variants, which satisfied the above QC procedures, from 287 CMUH and 63 TWB male gout patients and 671 TWB male controls. Supplementary Figure S1 shows that all subjects from CMUH and TWB share the same genetic background.

### Batch effect and population stratification correction by PCA

To adjust the effect that the integrated SNPs were from two resources and population stratification (different ethnic groups among Taiwanese, e.g. Fujian and Hakka), we performed PCA on SNPs with MAF >1% and satisfying the Hardy-Weinberg equilibrium QC. When plotted against PC9 and PC10, the SNPs of the two sites were finally mixed, so the first nine PCs were used for correction of the batch effects; PCA plots are in Supplementary Figure S2).

### Uncovering significant rare variants by ACAT-O

We applied the aggregated Cauchy association test (ACAT-O (Liu, et al., 2019); in R software to identify significant rare variants. ACATO is a set-based test that is particularly powerful when sparse causal variants exist in a variant set (window). ACAT-O first transforms the *p*-value of the burden test (Li and Leal, 2008; Madsen and Browning, 2009; Price, et al., 2010), SKAT test (Wu, et al., 2011), and ACAT-V (Liu, et al., 2019), to Cauchy variables. Then ACAT-O combines the above variables, each with two choices of weights, as the test statistic and evaluates the significance. We used the default Beta (1, 25) and Beta (1, 1) for the above two weights.

We split the 8,703,559 rare variants into ∼1.31 M windows of 4 kb with 2 kb overlaps in adjacent windows. In each window, we conducted ACAT-V, which implemented a logistic regression of gout status on all variants in the window conditional on covariates of age, uric acid levels, and the aforementioned nine PCs; similarly, the burden test and SKAT were also implemented. The significance threshold of ACAT-O was *p* < 3.8 × 10^−8^ after Bonferroni correction for multiple testing of ∼1.31 million windows.

### Statistical analysis

There are 682 rare variants (MAF < 1%) among the 61 windows uncovered to be significantly associated with gout susceptibility by ACAT-O (*P* < 3.8 × 10^−8^), and these RVs are deemed significant according to ACAT-O. For each significant rare variant, we computed the odds ratio and the associated 95% confidence interval, as well as the two-sample t-test for the equality of the proportion in cases and controls. Finally, logistic regression adjusted on serum urate levels was performed to evaluate the effects of rare variants on gout susceptibility.

## Results

### Study population

This study includes 350 male gout patients (287 from China Medical University Hospital (CMUH henceforth) and 63 from the Taiwan Biobank database (TWB henceforth)), whose data passed our QC procedures. In addition, it also included 671 male normal controls from TWB, which provided gout history, uric acid levels, demographic, and whole genome sequencing data. There were 40, 644, 135 and 42, 571, 357 SNPs called from the WGS data of CMUH and TWB, respectively. We intersected the SNP called from the two sites (CMUH_TWB) and subjected the 18, 426, 362 overlapping SNPs to quality check, of which 8,703,559 rare variants (MAF < 1%) satisfied the QC procedures; please see SNP genotyping and quality control in Materials and Methods for details.

The mean age of male gout patients and normal controls were 49.56 years (±15.99) and 49.69 years (±11.58), respectively, which was not significantly different (*P* = 0.888). There was too many mssing values of the covariate BMI for patients from CMUH, so BMI was not analyzed. However, uric acid of the two groups was significantly different (*P* < 2.2 × 10^−16^); see Supplementary Table S1 for details. In the subsequent analysis, serum urate was treated as a potential confounder of gout.

### Results of rare variant association tests

**FIGURE 1:**
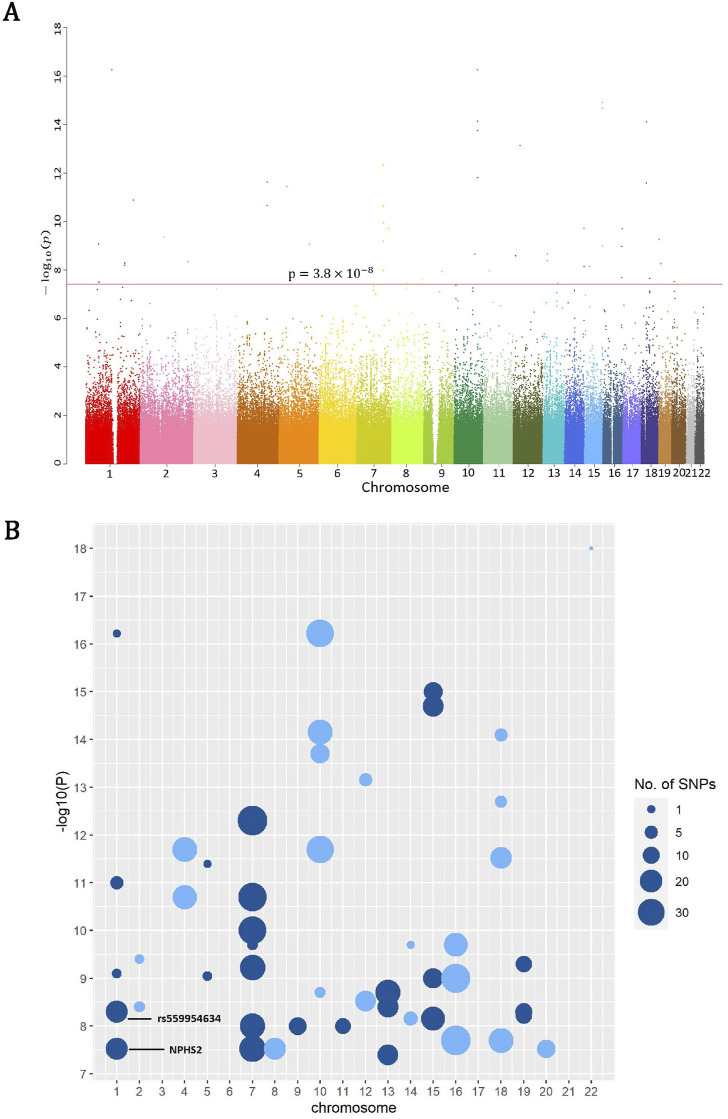
**(A)** The Manhattan plot depicted all *p*-values after the negative logarithm transformation of all ∼8.7 million RVs which passed QC across 22 pairs of autosomal chromosomes, where the red line corresponds to the threshold 3.8 × 10^−8^ (ACAT-O) for significant rare variants. **(B)** The log_10_ (1/*P*) of rare variants significantly associated with gout susceptibility show clusters in chromosomes 1, 7, 10, 16 and 18, where larger balls denote more variants in the corresponding windows.

The overall rare-variant analysis identified 61 windows significantly associated with gout disease (*p* < 3.8 × 10^−8^; ACAT-O), out of 1,314,794 windows analyzed. These windows consist of 682 distinct rare variants (Supplementary Table S2). The Manhattan plot depicted all *p*-values after the negative logarithm transformation of all ∼8.7 million RVs that passed QC across 22 pairs of autosomal chromosomes; note all RVs in a window share the same *p*-value from ACAT-O (Figure 1A). We further used balls with sizes proportional to the number of significant rare variants in each window (Figure 1B), and found significant RVs are clustered on chromosomes 1, 7, 10, 16, and 18.

Of the 682 significant rare variants, we further sifted these by CADD scores (Kircher, et al., 2014; Rentzsch, et al., 2019; van der Velde, et al., 2015) greater than 10; CADD is a widely used measure for variant deleteriousness that can prioritize causal variants effectively in genetic analysis, in particular highly penetrate contributors to severe Mendelian disorders. CADD is an annotation integrated from >60 genomic features, such as surrounding sequence context and gene model annotations. For any given variant, all the features are integrated into a CADD score which is a phred-scale rank score for all ∼9 billion potential single nucleotide variants (Rentzsch et al., 2019).

In particular, we identified rare variant rs559954634 (*p* = 6.4 × 10^−9^; ACAT-O; CADD = 13.5), and rs186763678 (*p* = 3.2 × 10^−8^; ACAT-O; CADD = 10.5) which is in intron of *NFIA*. *NFIA* is known to interact with *SLC2A9* that has the largest effect on serum urate levels. Gene-gene interaction *NFIA-SLC2A9* was reported to be significantly associated with serum urate in the Italian MICROS population (n = 1,201) and replicated in a Croatian population (n = 1772) (Wei, et al., 2011). The rare variant rs186763678 has an effect size of −1.79, odds ratio 0.24 (*P* = 0.176), and the proportion test of gout patients versus controls (*P* = 0.075) in this study.

The rare variant rs559954634 positively affects gout susceptibility (odds ratio = 4.85; *P* = 0.060); this is the first report. This variant has been annotated in dbSNP (Sherry, et al., 2001) but not in ClinVar (Landrum, et al., 2018) and GWAS (Uffelmann, et al., 2021). Table 1 consists of significant rare variants (*p* < 3.8 × 10^−8^; ACAT-O) with CADD score >10 or without CADD scores but |effect size| > 1.5, as WGS may reveal unannotated rare variants, so we also report the latter. Supplementary Table S3-1 summarizes their effect sizes, odds ratios, *P* values (the proportion test of gout patients versus normal controls, and logistic regression adjusted on serum urate), and CADD phred scores.

**TABLE 1:** The significant rare variants with CADD ≥10 or without CADD score but |effect size| ≥ 1.5 found in this study.

SNP name	Locus	Chr	Position	HGVS.c^a^	HGVS.p	Allele			CADD score
						MAF	Case	Control	
rs765272850	*FAM92B*	16	85,110,461	c.G21-1C	n.G21-1C	0.001	1	1	32.00
rs199599166	*FAM92B*	16	85,108,054	c.G301A	p.G101A	0.002	3	1	25.40
rs753091695	*FAM92B*	16	85,110,392	c.C89T	p.S30L	0.004	3	5	23.20
rs746009560	*OR7D4* *OR7E24*	19	9,214,314	c.C524T	p.P175L	0.001	1	1	21.80
rs75755442	*LINC00687*	20	11,878,042	c.C176 + 212T	NA	0.007	7	8	18.73
rs375422213	*CELF4* ^b^	18	39,025,897	NA	NA	0.001	1	2	18.03
rs1447484432	*CELF4* ^b^	18	39,026,967	NA	NA	0.001	1	1	17.52
rs932402166	*AC015660.5*	15	99,475,249	c.G248-442T	NA	0.002	3	2	17.24
rs77675394	*CELF4* ^b^	18	39,027,680	NA	NA	0.0005	1	0	17.10
rs144030951	*CELF4* ^b^	18	39,025,679	NA	NA	0.0005	1	0	16.56
rs57012644	*CELF4* ^b^	18	39,027,584	NA	NA	0.0005	1	0	16.06
rs1487893445	*SORCS1* ^b^	10	1,06,218,086	NA	NA	0.001	2	0	15.59
rs1485225053	*CELF4* ^b^	18	39,027,007	NA	NA	0.0005	1	0	15.41
rs1344344694	*LINC00687*	20	11,878,021	n.176 + 229_176 + 232del	NA	0.001	1	1	15.25
rs1258043800		12	10,489,523	NA	NA	0.001	0	3	14.68
rs541620279		12	10,492,869	NA	NA	0.001	1	1	14.44
rs950806939		18	39,027,469	NA	NA	0.001	1	1	14.30
**rs559954634** ^c^		1	178,632,863	NA	NA	0.003	5	2	13.48
rs369183475	AC105362.1	4	135,809,425	NA	NA	0.004	3	6	13.45
rs1323073113		15	99,534,543	NA	NA	0.001	0	3	12.93
rs75734921	*PLA2G4D*	15	42,094,432	c.C28A	p.P10T	0.0005	1	0	12.89
rs531158543		12	10,493,640	NA	NA	0.001	0	2	12.53
rs181190277		10	1,06,217,544	NA	NA	0.0005	1	0	11.91
rs1359426229		13	37,067,584	NA	NA	0.001	2	1	11.47
rs1475570291		12	10,492,576	NA	NA	0.002	1	3	11.18
rs375254150	C16orf95	16	87,170,326	n.C111-29223T	NA	0.004	3	6	11.03
rs745657928	AL162726.3	9	82,527,931	n.T247 + 31073A	NA	0.0005	1	0	10.74
rs77904948		12	10,489,582	NA	NA	0.002	1	3	10.65
**rs186763678** ^c^	*NFIA*	1	61,147,493	c.G559 + 58813T	n.G559 + 58813T	0.004	1	8	10.47
rs200909907		20	11,877,823	n.G176 + 431A	NA	0.001	1	1	10.25
rs148751159	*RALGPS2*	1	178,770,651	c.A-83-6031G	n.A-83-6031G	0.008	5	11	10.16
rs914948342		10	106,226,226	NA	NA	0.0005	1	0	10.01
—	*SLITRK6* ^b^	13	85,340,105	NA	NA	0.002	0	5	—
—	*SLITRK6* ^b^	13	85,340,778	NA	NA	0.003	6	1	—
—	*SLITRK6* ^b^	**13** ^c^	**85,340,782** ^c^	NA	NA	0.006	11	1	—

^a^ c. denotes coding DNA, reference sequence; n. denotes non-coding RNA, reference sequence. p denotes protein reference sequence; N + M denotes nucleotide M in the intron after (3′of) position N in the coding reference sequence; N-M denotes nucleotide M in the intron before (5′of) position N in the coding reference sequence; -N + M/-N-M denotes nucleotide in an intron in the 5′UTR; _ (underscore) denotes nucleotide numbering, used to indicate a range (e.g. in combination with a deletion, duplication, insertion, or variable sequence).

^b^ Genes not mapped in the merged window of the rare variants, but they are in the extended windows.

^c^ The novel RVs associated with gout in Taiwanese males revealed in this study.

Next, we merged adjacent significant windows of ACAT-O into larger ones and extended both edges of these windows upstream and downstream 1 Mb. Of these extended windows, the window of rs559954634 intersected with *NPHS2,* which is involved in serum urate and indirectly linked to gout through hyperuricemia. Moreover, *NPHS2* expresses in kidney tissues (queried from GTEx portal), and the kidneys play a major role in regulating uric acid levels. This suggests rs559954634 may be involved in gout, though further research is warranted. All overlapping genes in the 1 Mb-extended windows of rs559954634 are summarized in Supplementary Table S3-2. Moreover, there are 39 genes in the 1-Mb extended window of rs186763678; however, none is related to gout or serum urate, so we summarize these 39 genes in Supplementary Table S3-3.

### Prioritization of the rare variants in *NPHS2*

**FIGURE 2:**
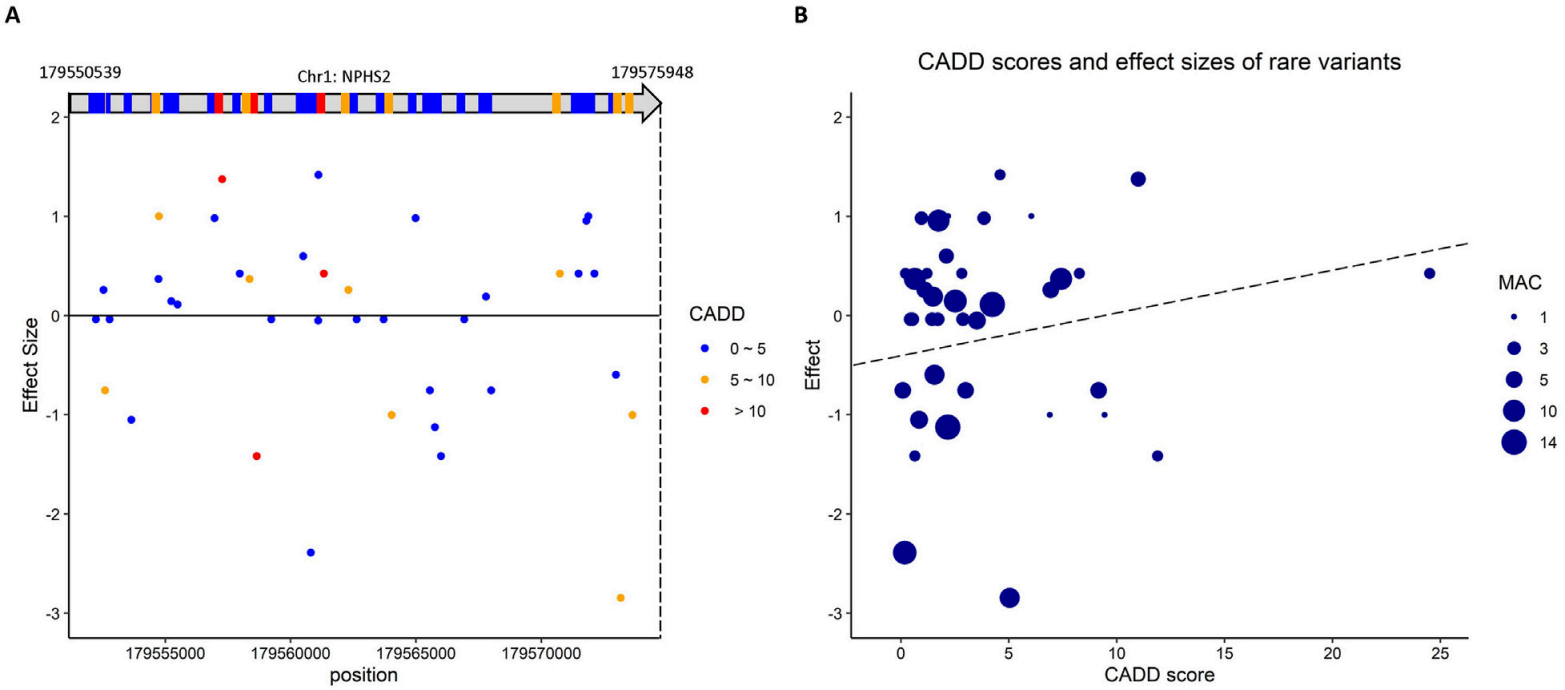
The putative rare variants in *NPHS2* which is involved in serum urate and indirectly linked to gout. **(A)** The scheme shows the positions of these variants in *NPHS2* and the corresponding effect sizes. **(B)** the rare variants of *NPHS2* with higher CADD scores are associated with larger positive effects on gout susceptibility.

Gene *NPHS2* encodes podocin, which is directly involved with serum urate levels (hyperuricemia; (Romi et al., 2017). Of the rare variants that passed the QC checks, 39 distinct rare variants are in *NPHS2*; however, none is significant in ACAT-O. Among these rare variants, rs202036853 (effect size = 5.79) shows a nominal association with gout adjusted on serum urate (*P* = 0.038; logistic regression) and has a CADD score of 11.0. Figure 2A shows the positions of these variants and the corresponding effect sizes. Figure 2B depicts that the rare variants of *NPHS2* with higher CADD scores are associated with more significant positive effects on gout susceptibility. The effect size, proportion test, logistic regression, and CADD score of these variants in *NPHS2* are in Supplementary Table S3-4.

### Another novel rare variant discovered

We also uncovered a novel rare variant 13-85340782-G-A (chr. 13; *P* = 3.5 × 10^−8^; ACAT-O), which has an odds ratio of 6.02 (*P* = 0.038) and is significant in the proportion test (*P* = 0.002). Two other RVs in the same window of 13-85340782-G-A also have |effect size| > 1.5, but their odds ratio for gout is insignificant (Supplementary Table S3-1). The RV 13-85340782-G-A is significantly associated with gout (*P* = 0.038; logistic regression), but the significance diminished when adjusted on serum urate (*P* = 0.124; logistic regression), which may indicate the association of this variant with gout is through serum urate. The 1 Mb-extended windows of this rare variant do not intersect with any known gene involved in gout or serum urate. The genes in this 1 Mb-extended window are in Supplementary Table S3-5.

### Rs559954634 and 13-85340782-G-A jointly are significantly associated with gout susceptibility

Recently, several genetic studies have reported that multiple rare variants play essential roles in complex genetic diseases, e.g., Alzheimer’s disease (Cruchaga et al., 2014), which support the “Common Disease Multiple Rare Variants” (CDMRVs) or “Common Disease Rare Variants” (CDRV) hypothesis (Schork et al., 2009). CDMRV and CDRV hypotheses argue that multiple rare variants and a single rare variant, each with relatively high penetrance, are the major contributors to common diseases, respectively. In this section, we applied multivariate logistic regression analysis to evaluate the joint effect of the rare variant in *NFIA* rs186763678, rs559954634, and 13-85340782-G-A discovered by ACAT-O and CADD scores/or effect size. The results support the “CDMRVs” hypothesis at *P <* 0.06 (multiple logistic regression). Specifically, univariate logistic regression analysis reveals that rs559954634 and 13-85340782-G-A are significantly associated with gout susceptibility (Table 2). When analyzed jointly, 13-85340782-G-A (beta = 1.80, *P =* 0.037; OR = 6.06) and rs559954634 (beta = 1.60, *P =* 0.057; OR = 4.93) are significantly associated with gout susceptibility at *P* < 0.06; the result supports CDMRVs at a non-stringent criteria.

**TABLE 2:** Logistic regression analysis of gout susceptibility on the three identified rare variants.

Univariate variable	$\hat{\beta }$	Odds ratio (95% CI)	*P* value
rs559954634	1.58	4.84 (1.04 – 33.97)	0.060
rs186763678	−1.44	0.24 (0.01 – 1.30)	0.176
13-85340782-G-A	1.80	6.02 (1.74 –89.68)	0.038

Multivariate Variable	$\hat{\beta }$	Odds ratio (95% CI)	*P* value
rs559954634	1.60	4.93 (1.06 – 34.56)	0.057
13-85340782-G-A	1.80	6.06 (1.75 – 90.45)	0.037

### Rare variants in known gout-related genes *ABCG2, SLC2A9* and *SLC22A12*

This study found 16 (16, 3) rare variants in the known gout-related gene ABCG2 (SLC2A9, SLC22A12). However, none of these are significant in ACAT-O; the smallest *P* values of these variants in *ABCG2, SLC2A9,* and *SLC22A12* are 0.004, 0.012, and 0.122, respectively (Supplementary Table S4-1). This may be due to our samples and those in previous studies being of different ancestry or our sample size not being large (under budget constraints). The position, allele frequency, and CADD score of these variants are summarized in Supplementary Table S4-2.

In the following, we report the rare variants in these genes, which have CADD scores ≥10 and are significant in both the odds ratio and the proportion test. The rare variant rs199897813 in *ABCG2* has a CADD score of 28.3, an effect size of 1.96, and an odds ratio equal to 9.71 (*P* = 0.038), respectively, and its proportion test of patients versus controls has *P* = 0.051. Moreover, three rare variants identified in *ABCG2,* rs548254708, rs34678167, and rs149106245, were previously reported in a Japanese cohort (Higashino et al., 2017), but neither is significant in odds ratio and the proportion test in this study; this is reasonable as individuals of these two cohorts are of different ancestry. Of the rare variant revealed in *SLC2A9,* rs150391338 has a CADD score of 10.4, effect size of −2.37, odds ratio 0.25 (*P* = 0.068), and it is significant in the proportion test (*p* = 0.018); detailed information is summarized in Supplementary Table S4-3.

### The rare variants in the remaining merged windows

We also computed the odds ratio and conducted the proportion test in the remaining merged windows of the significant rare variants (*P* < 3.8 × 10^−8^; ACAT-O). However, none of the rare variants therein satisfies both CADD ≥10 and is significant in odds ratio or the proportion test (*P* < 0.10).

## Discussion

Rare variants often contribute to complex diseases with large effect sizes per allele; however, the power to detect these variants remains limited (Chen et al., 2022). This study integrated data from male gout patients from CMUH and TWB and normal controls of TWB to identify 682 significant rare variants by ACAT-O. We further sifted these variants by the measure of variant deleteriousness CADD >10 (|effect size| > 1.5) to find rs559954634 and rs186763678 (13-85340782-G-A). These variants have been identified for the first time as associated with gout susceptibility in Taiwanese males. The carrier frequency of potentially pathogenic rs559954634 and 13-85340782-G-A is higher in gout patients when compared to normal controls (1.4% versus 0.30%; *P* = 0.046, and 3.1% versus 0.15%; *P* = 0.011; the proportion test). Conversely, the carrier frequency of potentially protective variant rs186763678 is higher in controls than in patients (0.29% versus 1.2%; *P* = 0.037; the proportion test). The identified potentially pathogenic (protective) RVs with a prevalence >1% in cases (controls) indicate that these RVs may be associated with gout in this population. Nevertheless, further studies are warranted, as the inflation factor *λ* of the ∼8.7 million QC-passed RVs is moderate (1.42).

In the neighborhood of rs559954634, we found *NPHS2* that encodes podocin, which is directly involved in hyperuricemia (Romi et al., 2017). Moreover, *NPHS2* is expressed in kidney tissues (queried from the GTEx portal), and the kidneys play a major role in regulating uric acid levels. This suggests that rs559954634 may be involved in gout, though further research is warranted. The rare variant rs186763678 is in the intron of *NFIA* which interacts with *SLC2A9,* while *SLC2A9* is known for lowering serum urate and protecting gout (Tin et al., 2018). The RV 13-85340782-G-A has an odds ratio of 6.06 (*P* = 0.037) and a large effect size (3.15).

We caution that these findings were statistically significant and prioritized by CADD scores, but the revealed RVs have not been verified biologically.

ACAT-O is a sliding-window-based method that uses the conventional 4 kb window size with 2 kb overlaps. Varying the window size to 5 kb (3 kb), we reran ACAT-O to yield the *p* value of rs559954634, rs186763678, and 13-85340782-G-A equal to 6.2 × 10^−9^, 3.15 × 10^−8^, and 3.4 × 10^−8^ (6.36 × 10^−9^, 3.15 × 10^−8^, and 3.40 × 10^−8^), respectively; all the three RVs remain significant for the ACAT-O (with 5 kb-windows) cutoff 4.7 × 10^−8^ (resulted from ∼1.06 million windows), but only rs559954634 is significant for the ACAT-O (with 3 kb-windows) cutoff 2.86 × 10^−8^ (resulted from ∼1.78 million windows). As the number of windows increases, e.g., prespecifying 3 kb for window size, the Bonferroni correction for multiple testing becomes too conservative, leading to power loss. Furthermore, it is noted in Li et al. (2019) that the sliding window methods are likely to lose power if the pre-specified window size is too big because it might include too many neutral variants, or if the pre-specified window size is too small that it might exclude adjacent regions containing association signals. To circumvent the difficulty of specifying a window size *a priori*, Li and colleagues introduced a dynamic scan procedure (SCANG) to flexibly detect the sizes and locations of RV association regions of WGS studies. Thus, applying SCANG to our data to yield robust RVs of gout in Taiwanese males is an interesting future research. As the number of subjects in this study is moderate, it will be valuable to further sequence whole genomes of male gout patients in Taiwan or analyze an independent cohort, such as the UK Biobank, to confirm/compare these unraveled RVs. Moreover, the combination of individual variants into a polygenic risk score (PRS) has the potential to be a predictor for a disease (Elliott et al., 2020); it may be helpful for the diagnosis of gout, in addition to increasing statistical power to detect genetic associations. Therefore, deriving a PRS using the identified 682 rare variants and integrating our data with WGS data of individuals of other ancestry, e.g., the UK Biobank, are also promising future research directions for gout study.

## Data Availability

The datasets presented in this article are not readily available because the data of 64 male gout patients and normal controls from TWB belongs to the Taiwan Biobank and can be accessed upon application at: https://www.biobank.org.tw/. The data of 321 male gout patients at CMUH are under IRB restriction and thus are not publicly available. Requests to access the datasets should be directed to the corresponding authors and with permission of the CMUH. However, WGS summary statistics for the CMUH-TWB data are available at https://staff.stat.sinica.edu.tw/gshieh/WGS-data.htm and at GWAS catalog ID: GCST90432173.
