# Supplementary material for: Whole-genome sequencing reveals rare variants associated with gout in Taiwanese males
Source: Front Genet. 2024 Sep 25;15:1423714. doi: 10.3389/fgene.2024.1423714 (PMC11462091; doi:10.3389/fgene.2024.1423714)
Supplement: Supplementary file 1 [file DataSheet2.PDF]

Table S1. clinical information of gout patients and normal controls in this study

Group	Gout cases	Controls	*P*-Value
n	350	671	
Age (s.e.)	49.56 (15.99)	49.69 (11.58)	0.888
Uric acid (s.e.)	8.62 (2.61)	6.30 (1.25)	$< 2.2\times10^{-16}$

Table S3-1. The results of significant rare variants (ACAT-O) with CADD > 10 or without CADD scores but |effect size| > 1.5.

SNP name	Allele Ref/Alt	Effect Size (allele)	Odds Ratio[a] (95% CI)	*P*-value		CADD score
				Odds Ratio	Proportion test (allele)	
rs765272850	C/G	0.424	1.92 (0.08, 48.65)	0.645	0.671	32.00
rs199599166	C/T	1.373	5.79 (0.74, 1.17E+02)	0.129	0.170	25.40
rs753091695	G/A	0.188	1.15 (0.24, 4.72)	0.847	0.851	23.20
rs746009560	G/A	0.424	1.92 (0.08, 48.65)	0.645	0.671	21.80
rs75755442	G/A	0.938	1.69 (0.59, 4.75)	0.314	0.349	18.73
rs375422213	G/T	-0.035	0.96 (0.04, 10.04)	0.972	0.972	18.03
rs1447484432	T/C	0.424	1.92 (0.08, 48.65)	0.645	0.671	17.52
rs932402166	C/A	1.041	2.89 (0.48, 22.03)	0.246	0.298	17.24
rs77675394	A/C	1.001	5.51E+05 (9.94E-24, NA)	0.968	0.317	17.10
rs144030951	GC/G	1.001	5.51E+05 (9.94E-24, NA)	0.968	0.317	16.56
rs57012644	G/A	1.001	5.51E+05 (9.94E-24, NA)	0.968	0.317	16.06
rs1487893445	T/G	1.416	1.50E+06 (2.30E-20, NA)	0.970	0.157	15.59
rs1485225053	A/T	1.001	5.51E+05 (9.94E-24, NA)	0.968	0.317	15.41

rs1344344694	ATAGT/A	0.424	1.92 (0.08, 48.65)	0.645	0.671	15.25
rs1258043800	A/G	-1.734	9.01E-07 (NA, 6.84E+22)	0.978	0.083	14.68
rs541620279	T/C	0.424	1.92 (0.08, 48.65)	0.645	0.671	14.44
rs950806939	G/A	0.424	1.92 (0.08, 48.65)	0.645	0.671	14.30
rs559954634	C/T	1.686	4.85 (1.04, 33.97)	0.060	0.092	13.48
rs369183475	T/G	-0.06	0.96 (0.20, 3.66)	0.952	0.952	13.45
rs1323073113	T/C	-1.734	9.01E-07 (NA, 6.84E+22)	0.978	0.083	12.93
rs75734921	G/T	1.001	5.51E+05 (9.94E-24, NA)	0.968	0.317	12.89
rs531158543	T/A	-1.415	2.45E-06 (NA, 1.60E+20)	0.973	0.157	12.53
rs181190277	T/C	1.001	5.51E+05 (9.94E-24, NA)	0.968	0.317	11.91
rs1359426229	T/C	0.982	3.85 (0.37, 83.03)	0.272	0.326	11.47
rs1475570291	A/G	-0.419	0.64 (0.03, 5.00)	0.698	0.675	11.18
rs375254150	G/A	-0.06	0.96 (0.20, 3.66)	0.952	0.952	11.03
rs745657928	T/C	1.001	5.51E+05 (9.94E-24, NA)	0.968	0.317	10.74
rs77904948	G/A	-0.419	0.64 (0.03, 5.00)	0.698	0.675	10.65
rs186763678	G/T	-1.784	0.24 (0.01, 1.30)	0.176	0.075	10.47
rs200909907	C/T	0.424	1.92 (0.08, 48.65)	0.645	0.671	10.25
rs148751159	A/G	-0.262	0.87 (0.27, 2.41)	0.797	0.793	10.16
rs914948342	T/G	1.001	5.51E+05 (9.94E-24, NA)	0.968	0.317	10.01
13-85340105-TTTA-T	TTTA/T	-2.240	8.98E-07 (NA, 5.79E+11)	0.972	0.025	-
13-85340778-G-A	G/A	2.197	3.92 (1.08, 55.05)	0.105	0.028	-
13-85340782-G-A	G/A	3.145	6.02 (1.74, 89.68)	0.038	0.002	-

[a]Odds Ratio = P(Gout=1)/P(Gout=0) for each variant

Table S3-2. The genes in the 1Mb-extended windows of the rare variant rs559954634.

Extended Range	No. of genes	Gene Name
1Mb	43	*AL513013.1, SETP10, AL160286.2, AL512326.2, AL365357.1, FAM20B, AL359075.1, MEF2AP1, TDRD5, ABL2, COX5BP8, AL137796.1, RASAL2-AS1,* ***NPHS2****, AL449106.1, RF00272, CRYZL2P, EIF4A1P11, RF00092, C1orf220, TOR3A, AL139132.1, MIR4424, AXDND1, RASAL2, SOAT1, SEC16B, CRYZL2P-SEC16B, AL160281.1, PTPN2P1, AL512326.1, RF00272, ANGPTL1, RALGPS2, TEX35, AL512326.3, CLEC20A, HNRNPA1P54, RF00017, AL160286.3, LINC01741, AL160286.1, RNU5F-2P, RNA5SP69*

Table S3-3. The genes in the 1Mb-extended windows of the rare variant rs186763678

Extended range	No. of genes	Gene Name
1Mb	39	*RN7SL180P, RNU6-1177P, MIR3116-1, PIGPP2, USP1, DOCK7, RF00019, AC099794.1, LINC01748, RNU6-371P, AL138847.2, RPS15AP7, NFIA, AL162739.1, RNU6-414P, NFIA-AS2, L1TD1, AC099791.3, AC099794.2, TM2D1, MIR3116-2, PATJ, AC099791.2, ANGPTL3, AC096543.1, AL451044.1, ATG4C, AC096947.1, AC099791.1, LAMTOR5P1, KANK4, AL162400.1, AC103923.1, LINC01739, NFIA-AS1, AC097066.1, AL138847.1, AL162400.2, AC099792.1*

Table S3-4. The results of rare variants in *NPHS2* (effect size, odds ratio, proportion test, logistic regression with serum urate, and ACAT-O)

SNP name	Effect Size	Odds ratio (95% CI)	Odds Ratio *p*-value	*P*-values			CADD score
				Proportion test	Logistic regression with serum urate	ACAT-O	
rs1294075077	-0.035	0.958 (0.044, 10.04)	0.972	0.972	0.781	0.780	1.435
rs1279901898	0.260	1.28 (0.168, 7.758)	0.788	0.795	0.660	0.780	1.106
1:179552593:G:A	-0.753	0.478 (0.024, 3.245)	0.510	0.451	0.973	0.780	9.159
rs572403412	-0.035	0.958 (0.044, 10.04)	0.972	0.972	0.868	0.780	2.878
rs554374156	-1.050	0.382 (0.02, 2.378)	0.380	0.294	0.477	0.780	0.841
rs148224383	0.369	1.281 (0.326, 4.515)	0.702	0.712	0.418	0.394	0.652
rs972907553	1.001	551148.9 (0, NA)	0.968	0.317	0.979	0.394	6.050
rs1003632356	0.146	1.097 (0.286, 3.657)	0.884	0.884	0.942	0.394	2.524
rs368723845	0.114	1.066 (0.325, 3.11)	0.909	0.909	0.465	0.394	4.235
rs1162773109	0.983	3.851 (0.368, 83.03)	0.272	0.326	0.197	0.297	3.851
**rs202036853**	1.376	5.793 (0.739, 117.339)	0.129	0.170	**0.039**	0.297	11.000
rs1305679886	0.425	1.92 (0.076, 48.645)	0.645	0.671	0.931	0.297	0.215
rs1385123145	0.369	1.281 (0.326, 4.515)	0.702	0.712	0.756	0.297	7.429
rs571849554	-1.416	0 (NA, 1.6E+20)	0.973	0.157	0.981	0.297	11.900
rs371450814	-0.035	0.958 (0.044, 10.04)	0.972	0.972	0.482	0.297	1.464

	Effect	Odds ratio (95% CI)	Odds	*P*-value			

SNP name	Effect Size	Odds ratio (95% CI)	Odds ratio *p*-value	*P*-value: Proportion test	*P*-value: Logistic regression with serum urate	*P*-value: ACAT-O	CADD score
continued							
rs1371917639	0.601	1.922 (0.23, 16.078)	0.514	0.548	0.747	0.450	2.112
rs141837383	-2.387	0.172 (0.009, 0.889)	0.092	0.017	0.220	0.450	0.180
rs144292137	-0.049	0.958 (0.132, 4.935)	0.961	0.961	0.882	0.450	3.523
rs1351435115	1.418	1502488 (0, NA)	0.970	0.157	0.974	0.450	4.592
rs200437667	0.425	1.92 (0.076, 48.645)	0.645	0.671	0.461	0.450	24.500
rs1281815042	0.260	1.28 (0.168, 7.758)	0.788	0.795	0.660	0.501	6.945
rs1294790856	-0.035	0.958 (0.044, 10.04)	0.972	0.972	0.174	0.501	0.533
rs189485234	-0.035	0.958 (0.044, 10.04)	0.972	0.972	0.791	0.501	1.703
rs144600350	-1.001	0 (NA, 3.7E+23)	0.971	0.317	0.981	0.267	9.437
rs940134538	0.983	3.851 (0.368, 83.03)	0.272	0.326	0.306	0.267	0.947
rs1286629019	-0.753	0.478 (0.024, 3.245)	0.510	0.451	0.603	0.267	0.086
rs182880628	-1.125	0.519 (0.117, 1.674)	0.316	0.261	0.548	0.267	2.173
rs1386090831	-1.416	0 (NA, 1.6E+20)	0.973	0.157	0.979	0.267	0.649
rs556977242	-0.035	0.958 (0.044, 10.04)	0.972	0.972	0.712	0.267	0.448
rs545788840	0.193	1.152 (0.235, 4.721)	0.847	0.847	0.968	0.267	1.482
rs568264857	-0.753	0.478 (0.024, 3.245)	0.510	0.451	0.215	0.267	3.000
rs562400811	0.425	1.92 (0.076, 48.645)	0.645	0.671	0.695	0.149	8.270
rs1342315537	0.425	1.92 (0.076, 48.645)	0.645	0.671	0.706	0.149	1.212
rs1175010496	0.955	1.93 (0.534, 6.984)	0.301	0.340	0.755	0.149	1.748

SNP name	Size		Ratio p-value	Proportion test	Logistic regression with serum urate	ACAT-O	CADD score
continued							
rs1196446876	1.001	551148.9 (0, NA)	0.968	0.317	0.982	0.149	2.187
rs567457665	0.425	1.92 (0.076, 48.645)	0.645	0.671	0.334	0.149	2.827
rs560972021	-0.595	0.637 (0.093, 2.781)	0.582	0.552	0.721	0.149	1.553
1:179573167:A:C	-2.845	0 (NA, 272716.260)	0.964	0.005	0.974	0.149	5.041
1:179573635:C:T	-1.001	0 (NA, 3.7E+23)	0.971	0.317	0.983	0.149	6.898

Table S3-5. The genes in the 1Mb-extended windows of the rare variant 13-85340782-G-A

Extended range	No. of genes	Gene Name
1Mb	12	*MTND4P1, MTND5P3, LINC00333, LINC00375, LINC00351, AL356413.1, SLITRK6, AL162373.1, MOB1AP1, AL354994.1, FO624990.1, DDX6P2*

Table S4-1. The result of the gout-related genes *ABCG2, SLC2A9,* and *SLC22A12,* which are not significant in ACAT-O, but their windows have maximum CADD score > 10

window	No. of variants in the window	*P*-Values							CADD score
		ACAT-O	SKAT (1,25)	SKAT (1,1)	Burden (1,25)	Burden (1,1)	ACAT-V (1,25)	ACAT-V (1,1)	
ABCG2									
4:88128001-88132000	10	0.704	0.273	0.295	0.620	0.657	0.879	0.886	41.00
4:88130001-88134000	22	1.000	0.592	0.637	1.000	1.000	0.875	0.880	41.00
4:88092001-88096000	8	0.219	0.134	0.130	0.389	0.437	0.232	0.240	37.00
4:88094001-88098000	11	0.086	0.185	0.189	0.067	0.068	0.067	0.068	37.00
4:88096001-88100000	14	0.031	0.287	0.318	0.013	0.016	0.032	0.049	28.00
4:88098001-88102000	16	0.057	0.294	0.304	0.022	0.030	0.081	0.108	28.00
4:88116001-88120000	12	0.640	0.400	0.376	0.149	0.142	0.915	0.922	26.40
4:88118001-88122000	11	0.743	0.200	0.211	0.480	0.433	0.926	0.930	26.40
4:88120001-88124000	18	0.825	0.389	0.405	0.583	0.602	0.935	0.937	23.60
4:88136001-88140000	10	1.000	1.000	1.000	0.888	1.000	0.901	0.899	22.50
4:88138001-88142000	13	1.000	0.441	0.454	1.000	1.000	0.700	0.720	22.50
4:88140001-88144000	14	1.000	0.241	0.283	1.000	1.000	0.811	0.822	15.68
4:88112001-88116000	7	1.000	1.000	1.000	1.000	1.000	0.908	0.911	13.47
4:88114001-88118000	12	1.000	1.000	1.000	0.911	1.000	0.922	0.924	13.47
4:88142001-88146000	12	1.000	0.273	0.311	1.000	1.000	0.884	0.887	11.62
ABCG2(cont.)									
4:88144001-88148000	16	0.697	0.765	0.755	0.530	0.630	0.709	0.722	11.62
4:88156001-88160000	8	1.000	1.000	1.000	1.000	1.000	0.656	0.647	10.29

window	No. of variants in the window	*P*-Values							CADD score
		ACAT-O	SKAT (1,25)	SKAT (1,1)	Burden (1,25)	Burden (1,1)	ACAT-V (1,25)	ACAT-V (1,1)	
4:88158001-88162000	9	1.000	0.538	0.504	1.000	1.000	0.386	0.377	10.29
4:88172001-88176000	9	0.156	0.123	0.135	0.245	0.252	0.128	0.135	10.22
4:88174001-88178000	15	0.004	0.011	0.015	0.049	0.048	0.001	0.002	10.22
4:88214001-88218000	8	1.000	0.583	0.547	1.000	1.000	0.349	0.372	10.19
4:88216001-88220000	9	0.460	0.614	0.592	0.375	0.370	0.412	0.407	10.19
4:88198001-88202000	13	0.448	0.806	0.793	0.245	0.234	0.293	0.279	10.07
4:88200001-88204000	8	0.343	0.741	0.755	0.125	0.130	0.411	0.444	10.07
4:88176001-88180000	20	1.000	0.009	0.010	1.000	1.000	0.001	0.002	10.01
4:88178001-88182000	17	0.197	0.072	0.081	0.310	0.300	0.658	0.684	10.01
SLC2A9									
4:9824001-9828000	19	1.000	0.713	0.650	1.000	1.000	0.364	0.356	34.00
4:9826001-9830000	13	0.415	0.510	0.474	0.388	0.367	0.398	0.367	34.00
4:9780001-9784000	23	1.000	0.500	0.465	1.000	1.000	0.255	0.248	27.10
4:9782001-9786000	22	1.000	0.643	0.607	1.000	1.000	0.366	0.351	27.10
4:9790001-9794000	15	0.938	0.979	0.970	0.855	0.861	0.613	0.600	16.54
SLC2A9(cont.)									
4:9792001-9796000	15	0.808	0.745	0.753	0.883	0.921	0.530	0.524	16.54
4:9912001-9916000	11	0.021	0.226	0.295	0.008	0.011	0.031	0.047	16.08
4:9914001-9918000	14	0.292	0.602	0.609	0.123	0.129	0.416	0.428	16.08
4:9966001-9970000	16	0.216	0.579	0.552	0.087	0.087	0.377	0.397	15.90
4:9968001-9972000	14	1.000	0.589	0.516	1.000	1.000	0.316	0.311	15.90
4:9810001-9814000	14	0.576	0.815	0.811	0.363	0.376	0.390	0.403	14.76
4:9812001-9816000	11	1.000	1.000	1.000	0.906	0.850	0.910	0.907	14.76

window	No. of variants in the window	*P*-Values							CADD score
		ACAT-O	SKAT (1,25)	SKAT (1,1)	Burden (1,25)	Burden (1,1)	ACAT-V (1,25)	ACAT-V (1,1)	
4:9852001-9856000	12	0.398	0.318	0.317	0.609	0.536	0.339	0.324	13.70
4:9854001-9858000	9	0.937	0.452	0.398	0.258	0.234	0.990	0.591	13.70
4:9990001-9994000	12	0.465	0.667	0.679	0.357	0.376	0.347	0.365	13.37
4:9992001-9996000	13	0.634	0.780	0.788	0.519	0.528	0.507	0.513	13.37
4:9844001-9848000	11	0.397	0.449	0.362	0.451	0.404	0.409	0.321	11.35
4:9846001-9850000	16	0.786	0.622	0.653	0.303	0.298	0.921	0.925	11.35
4:10030001-10034000	19	0.224	0.367	0.317	0.526	0.469	0.105	0.101	11.25
4:10032001-10036000	15	0.217	0.362	0.307	0.280	0.243	0.142	0.131	11.25
4:9778001-9782000	24	0.742	0.814	0.818	0.654	0.706	0.688	0.690	10.89
4:9978001-9982000	11	0.708	0.870	0.936	0.287	0.307	0.324	0.340	10.77
4:9980001-9984000	13	0.605	0.869	0.871	0.309	0.311	0.327	0.329	10.77
SLC2A9(cont.)									
4:9884001-9888000	15	1.000	0.432	0.439	1.000	1.000	0.933	0.948	10.65
4:9886001-9890000	17	1.000	0.427	0.420	1.000	1.000	0.682	0.672	10.65
4:9868001-9872000	13	1.000	0.983	1.000	1.000	1.000	0.899	0.897	10.41
4:9870001-9874000	12	1.000	0.910	1.000	1.000	1.000	0.968	0.939	10.41
4:9838001-9842000	11	0.497	0.684	0.682	0.347	0.375	0.420	0.451	10.38
4:9840001-9844000	7	0.511	0.663	0.677	0.302	0.291	0.578	0.580	10.38
4:9818001-9822000	18	0.485	0.578	0.547	0.415	0.396	0.489	0.485	10.29
4:9820001-9824000	20	0.636	0.386	0.415	0.692	0.635	0.765	0.771	10.29
SLC22A12									
11:64598001-64602000	17	1.000	0.985	0.996	1.000	1.000	0.941	0.943	22.80

window	No. of variants in the window	*P*-Values							CADD score
		ACAT-O	SKAT (1,25)	SKAT (1,1)	Burden (1,25)	Burden (1,1)	ACAT-V (1,25)	ACAT-V (1,1)	
11:64596001-64600000	13	0.913	0.795	0.796	0.524	0.505	0.979	0.908	10.67

Table S4-2. The information of rare variants in known gout-related genes *ABCG2, SLC2A9,* and *SLC22A12,* which are not significant by ACAT-O, but their SNPs have maximum CADD score >10

The criteria to select the variants are (1) CADD score ≥ 15, (2) 10 ≤ CADD score ≤ 15, odds ratio p-value or proportion test p-value < 0.10, and (3) logistic regression (conditional on serum urate) p-value < 0.05

				Allele				
SNP name	Chr	Locus	Position	ref/alt	Freq.	case	control	CADD Score
ABCG2								
rs72552713	4	*ABCG2*	88131805	G/A	0.004	3	5	41.00
rs548254708	4	*ABCG2*	88095534	G/A	0.000	0	1	37.00
rs199897813	4	*ABCG2*	88094576	C/T	0.003	5	1	28.30
rs1444855501	4	*ABCG2*	88099428	T/C	0.000	1	0	28.00
rs569617981	4	*ABCG2*	88095539	A/T	0.000	1	0	27.50
rs34678167	4	*ABCG2*	88118145	G/A	0.003	1	5	26.40
rs149106245	4	*ABCG2*	88131209	T/A	0.007	3	11	25.60
rs528655917	4	*ABCG2*	88131113	C/T	0.004	4	4	25.20
rs762937915	4	*ABCG2*	88121773	C/T	0.001	1	2	23.60
rs767611090	4	*ABCG2*	88121736	T/C	0.000	1	0	23.00
rs761288842	4	*ABCG2*	88139863	G/C	0.001	1	1	22.50
rs779737784	4	*ABCG2*	88140059	A/T	0.001	1	1	15.68
rs544499758	4	*ABCG2*	88097563	G/C	0.001	2	1	15.58
rs192958180	4	*ABCG2*	88145183	A/C	0.001	0	3	11.62
rs1391665588	4	*ABCG2*	88178607	A/G	0.004	7	2	6.06
rs1481594016	4	*ABCG2*	88164415	G/C	0.005	8	2	3.58
rs535846073	4	*ABCG2*	88227071	G/A	0.010	13	7	1.07

SLC2A9								
rs752823281	4	*SLC2A9*	9826457	G/T	0.000	0	1	34.00
rs778635010	4	*SLC2A9*	9783083	T/C	0.001	1	1	27.10
rs557890668	4	*SLC2A9*	9782934	T/C	0.001	2	0	25.80
rs752776884	4	*SLC2A9*	9782817	A/G	0.001	1	1	25.40
-	4	*SLC2A9*	9782838	A/G	0.001	1	1	23.50
rs181755864	4	*SLC2A9*	9793220	A/T	0.004	2	6	16.54
rs746600036	4	*SLC2A9*	9782893	CAAG/C	0.000	1	0	16.24
rs1426140739	4	*SLC2A9*	9915591	A/G	0.001	1	2	16.08
-	4	*SLC2A9*	9969180	A/G	0.000	0	1	15.90
rs150391338	4	*SLC2A9*	9840248	A/C	0.008	2	15	10.38
rs140148860	4	*SLC2A9*	9821928	A/G	0.007	2	12	10.29
rs34788376	4	*SLC2A9*	9997430	A/G	0.009	10	8	2.06
rs186345821	4	*SLC2A9*	9965464	G/T	0.010	13	7	0.97
rs146744389	4	*SLC2A9*	9938135	C/G	0.005	6	4	0.59
rs148071147	4	*SLC2A9*	9792390	G/A	0.007	11	4	0.53
rs1232915724	4	*SLC2A9*	10029943	AT/A	0.003	3	3	0.34
SLC22A12								
rs200104135	11	*SLC22A12*	64600740	C/T	0.003	0	6	22.80
rs1432222711	11	*SLC22A12*	64598451	G/A	0.001	0	3	10.67
rs376000964	11	*SLC22A12*	64594646	G/C	0.004	5	3	2.20

Table S4-3. The results of rare variants in gout-related genes *ABCG2, SLC2A9,* and *SLC22A12,* with effect size, odds ratio, proportion test, logistic regression, and CADD score ≥ 10.

The criteria to select the variants are: (1) CADD score ≥ 15, (2) 10 ≤ CADD score ≤ 15, odds ratio p-value or proportion test p-value < 0.10, or (3) Logistic regression (conditional on serum urate) p-value ≤ 0.05

SNP name	Effect Size	Odds Ratio (95% CI)	Odds Ratio *P*-Value	*P*-value			CADD score
				Proportion test (sample)	Proportion test (allele)	Logistic regression (with serum urate)	
ABCG2							
rs72552713	0.188	1.15 (0.24, 4.72)	0.847	0.847	0.851	0.413	41.00
rs548254708	-1.000	6.68E-06 (NA, 3.70E+23)	0.971	0.317	0.317	0.983	37.00
rs199897813	1.957	9.71 (1.56, 1.86E+02)	0.038	0.050	0.051	0.097	28.30
rs1444855501	1.001	5.51E+05 (9.94E-24, NA)	0.968	0.317	0.317	0.980	28.00
rs569617981	1.001	5.51E+05 (9.94E-24, NA)	0.968	0.317	0.317	0.981	27.50
rs34678167	-1.048	0.38 (0.02, 2.38)	0.380	0.294	0.295	0.535	26.40
rs149106245	-1.122	0.52 (0.12, 1.67)	0.316	0.261	0.262	0.527	25.60
rs528655917	0.850	1.93 (0.45, 8.20)	0.355	0.394	0.395	0.079	25.20
rs762937915	-0.035	0.96 (0.04, 10.04)	0.972	0.972	0.972	0.555	23.60
rs767611090	1.001	5.51E+05 (9.94E-24, NA)	0.968	0.317	0.317	0.979	23.00
rs761288842	0.424	1.92 (0.08, 48.65)	0.645	0.671	0.671	0.525	22.50
rs779737784	0.424	1.92 (0.08, 48.65)	0.645	0.671	0.671	0.723	15.68
rs544499758	0.982	3.85 (0.37, 83.03)	0.272	0.326	0.326	0.583	15.58
rs192958180	-1.734	9.01E-07 (NA, 6.84E+22)	0.978	0.083	0.083	0.978	11.62

SNP name	Effect Size	Odds Ratio (95% CI)	Odds Ratio *P*-Value	*P*-Value			CADD score
				Proportion test (sample)	Proportion test (allele)	Logistic regression (with serum urate)	
ABCG2 (cont.)							
rs1391665588	2.179	6.83 (1.64, 45.98)	0.017	0.029	0.030	0.005	6.06
rs1481594016	2.393	7.82 (1.95, 52.03)	0.010	0.017	0.017	0.006	3.58
rs535846073	2.442	3.66 (1.48, 9.82)	0.006	0.014	0.015	0.003	1.07
SLC2A9							
rs752823281	-1.000	6.68E-06 (NA, 3.70E+23)	0.971	0.317	0.317	0.982	34.00
rs778635010	0.424	1.92 (0.08, 48.65)	0.645	0.671	0.671	0.874	27.10
rs557890668	1.416	1.50E+06 (2.30E-20, NA)	0.970	0.157	0.157	0.977	25.80
rs752776884	0.424	1.92 (0.08, 48.65)	0.645	0.671	0.671	0.174	25.40
4:9782838:A:G	0.424	1.92 (0.08, 48.65)	0.645	0.671	0.671	0.351	23.50
rs181755864	-0.594	0.64 (0.09, 2.78)	0.582	0.552	0.553	0.639	16.54
rs746600036	1.001	5.51E+05 (9.94E-24, NA)	0.968	0.317	0.317	0.984	16.24
rs1426140739	-0.035	0.96 (0.04, 10.04)	0.972	0.972	0.972	0.829	16.08
4:9969180:A:G	-1.000	6.68E-06 (NA, 3.70E+23)	0.971	0.317	0.317	0.982	15.90
rs150391338	-2.372	0.25 (0.04, 0.90)	0.068	0.017	0.018	0.194	10.38
rs140148860	-1.862	0.32 (0.05, 1.17)	0.133	0.062	0.063	0.308	10.29
rs34788376	1.681	2.44 (0.95, 6.44)	0.063	0.091	0.093	0.017	2.06
rs186345821	2.442	3.32 (1.38, 8.82)	0.010	0.023	0.015	0.029	0.97
rs146744389	1.476	2.91 (0.83, 11.44)	0.100	0.139	0.140	0.020	0.59

SNP name	Effect Size	Odds Ratio (95% CI)	Odds Ratio *P*-value	Proportion test (sample)	*P*-value		CADD score
					Proportion test (allele)	Logistic regression (with serum urate)	
SLC2A9(cont.)							
rs148071147	2.583	5.41 (1.84, 19.64)	0.004	0.010	0.010	0.010	0.53
rs1232915724	0.736	1.93 (0.35, 10.45)	0.424	0.461	0.462	0.024	0.34
SLC22A12							
rs200104135	-2.455	8.97E-07 (NA, 9.20E+08)	0.969	0.014	0.014	0.973	22.80
rs1432222711	-1.734	9.01E-07 (NA, 6.84E+22)	0.978	0.083	0.083	0.980	10.67
rs376000964	1.429	3.23 (0.79, 15.81)	0.110	0.152	0.153	0.044	2.20
