# Supplementary material for: Whole-genome sequencing reveals rare variants associated with gout in Taiwanese males
Source: Front Genet. 2024 Sep 25;15:1423714. doi: 10.3389/fgene.2024.1423714 (PMC11462091; doi:10.3389/fgene.2024.1423714)
Supplement: Supplementary file 2 [file DataSheet3.PDF]

Table S2. The information of the 682 significant rare variants associated with gout susceptibility identified by ACAT-O ($P < 3.8\times10^{-8}$).

SNP name	Chr	Gene name	Position	Allele				ACAT-O p-value
				ref/alt	Freq.	case	control	
rs1372698731	10		106212478	A/G	0.001	1	1	5.55E-17
rs7919865	10		106214091	G/A	0.000	1	0	5.55E-17
rs568426587	10		106214220	G/A	0.000	1	0	5.55E-17
	10		106214339	T/G	0.000	1	0	5.55E-17
rs7923787	10		106215049	A/G	0.000	1	0	5.55E-17
rs7924257	10		106215138	G/T	0.000	1	0	5.55E-17
rs11445512	10		106215143	T/TA	0.000	1	0	5.55E-17
rs10884270	10		106215357	G/C	0.000	1	0	5.55E-17
rs1326346	10		106215677	C/T	0.000	1	0	5.55E-17
rs7919468	10		106214079	A/G	0.000	1	0	5.55E-17
rs6584732	10		106214049	C/T	0.000	1	0	5.55E-17
rs1204886078	10		106217254	T/A	0.001	0	2	5.55E-17
rs79999861	10		106217730	T/C	0.001	2	0	5.55E-17
rs1487893445	10		106218086	T/G	0.001	2	0	5.55E-17
rs185784242	10		106215042	A/G	0.006	6	7	5.55E-17
rs1393690205	10		106216683	G/A	0.001	1	2	5.55E-17
rs1326345	10		106215720	A/T	0.000	1	0	5.55E-17

rs573853015	10	106216102	C/T	0.001	1	2	5.55E-17
rs182647717	10	106219389	G/C	0.003	2	4	5.55E-17
rs1288585242	10	106212677	T/A	0.000	1	0	5.55E-17
rs1481311350	10	106212989	A/G	0.000	1	0	5.55E-17
rs3850677	10	106213515	T/A	0.000	1	0	5.55E-17
rs6584730	10	106213626	C/A	0.000	1	0	5.55E-17
rs1326344	10	106215893	T/C	0.000	1	0	5.55E-17
rs11599541	10	106218545	T/C	0.000	1	0	5.55E-17
rs10786926	10	106218614	G/A	0.000	1	0	5.55E-17
rs543214990	10	106218823	G/A	0.000	1	0	5.55E-17
rs1999354	10	106218963	G/A	0.000	1	0	5.55E-17
rs1999355	10	106219044	A/T	0.000	1	0	5.55E-17
rs1999356	10	106219128	T/A	0.000	1	0	5.55E-17
rs1999357	10	106219135	A/G	0.000	1	0	5.55E-17
rs755823356	10	106218378	G/A	0.000	1	0	5.55E-17
rs11192769	10	106219343	C/T	0.000	1	0	5.55E-17
rs11192771	10	106219770	G/C	0.000	1	0	5.55E-17
rs11192772	10	106219875	T/C	0.000	1	0	5.55E-17
rs747491542	10	106213702	C/A	0.001	1	1	5.55E-17
rs147524852	10	106218218	C/G	0.007	5	10	5.55E-17
rs1429802969	10	106219760	A/G	0.002	2	3	5.55E-17
rs148113742	10	106214291	G/C	0.005	3	7	5.55E-17
rs11192770	10	106219748	G/T	0.000	1	0	5.55E-17

rs7922310	10	106217925	T/C	0.000	1	0	5.55E-17
rs11192768	10	106217696	G/A	0.000	1	0	5.55E-17
rs12765942	10	106216018	T/C	0.000	1	0	5.55E-17
rs7917870	10	106216804	T/A	0.000	1	0	5.55E-17
rs7917874	10	106216819	T/C	0.000	1	0	5.55E-17
rs7901506	10	106216875	G/A	0.000	1	0	5.55E-17
rs11192765	10	106217042	C/T	0.000	1	0	5.55E-17
rs11192766	10	106217110	C/T	0.000	1	0	5.55E-17
rs11192767	10	106217320	C/T	0.000	1	0	5.55E-17
rs181190277	10	106217544	T/C	0.000	1	0	5.55E-17
rs565744460	10	106218220	A/C	0.003	3	3	5.55E-17
rs183212772	10	106215232	C/G	0.003	3	4	5.55E-17
rs1375124261	10	106215609	C/A	0.002	2	2	5.55E-17
rs187505547	10	106216229	G/C	0.001	1	1	5.55E-17
rs1362069355	1	120085671	G/GGTCTTC	0.001	3	0	5.55E-17
rs1383183169	10	106214103	T/C	0.001	1	1	5.55E-17
rs375759502	10	106217187	C/T	0.001	1	1	5.55E-17
rs532115281	15	99471157	G/A	0.004	2	6	1.22E-15
rs369381438	15	99473393	A/G	0.001	1	1	1.22E-15
rs186087050	15	99473416	G/A	0.007	10	4	1.22E-15
rs984216585	15	99471386	G/A	0.001	1	1	1.22E-15
rs148062668	15	99472838	C/T	0.004	4	5	1.22E-15
rs1489632562	15	99471074	C/G	0.006	3	9	1.22E-15

rs994553954	15	99471586	G/A	0.002	3	1	1.22E-15
rs1254613418	15	99473501	T/C	0.001	2	1	1.22E-15
	15	99470496	C/G	0.000	0	1	1.22E-15
rs1371977410	15	99473107	T/C	0.000	0	1	1.22E-15
rs183693481	15	99471468	T/C	0.001	1	2	1.22E-15
rs1337659370	15	99470595	G/A	0.003	2	5	1.22E-15
rs77349979	15	99472241	A/G	0.001	2	0	1.22E-15
rs751965731	15	99474566	C/T	0.001	1	2	2.16E-15
rs1219021646	15	99474565	G/A	0.001	1	2	2.16E-15
rs932402166	15	99475249	C/A	0.002	3	2	2.16E-15
rs905741332	15	99475787	A/G	0.004	4	5	2.16E-15
rs558888439	15	99475580	T/A	0.001	0	2	2.16E-15
rs745669887	15	99475641	T/C	0.001	1	2	2.16E-15
rs74035786	15	99474712	C/T	0.002	1	3	2.16E-15
rs187883489	15	99474498	C/G	0.009	9	10	2.16E-15
rs139945702	15	99474300	C/T	0.005	4	6	2.16E-15
rs566279945	15	99475143	C/T	0.002	0	5	2.16E-15
rs377761768	15	99474003	G/A	0.009	6	12	2.16E-15
rs1003898497	10	106220672	G/A	0.001	1	2	7.22E-15
rs1432695973	10	106220671	C/T	0.001	1	2	7.22E-15
rs12244862	10	106221189	T/C	0.000	1	0	7.22E-15
rs12266500	10	106220937	G/C	0.000	1	0	7.22E-15
rs11192774	10	106220692	T/C	0.000	1	0	7.22E-15

rs543901845	10		106220117	T/C	0.001	1	1	7.22E-15
rs59555021	10		106220461	AC/A	0.000	1	0	7.22E-15
rs12266576	10		106221071	G/A	0.000	1	0	7.22E-15
rs377591132	18	ANKRD29	23627816	T/A	0.001	1	1	7.83E-15
rs6507792	18	ANKRD29	23629736	G/T	0.001	2	0	7.83E-15
rs117447896	18	ANKRD29	23626288	T/G	0.001	2	0	7.83E-15
	18	ANKRD29	23626924	C/T	0.000	0	1	7.83E-15
rs59371064	18	ANKRD29	23626859	G/C	0.001	2	0	7.83E-15
rs73355621	10		106224755	A/G	0.000	1	0	1.76E-14
rs77657773	10		106224509	C/T	0.000	1	0	1.76E-14
rs9919496	10		106225967	C/G	0.000	1	0	1.76E-14
rs1853960	10		106225513	C/T	0.000	1	0	1.76E-14
rs1389350601	10		106225670	C/A	0.000	1	0	1.76E-14
rs73355615	10		106224579	C/T	0.000	1	0	1.76E-14
rs34438286	10		106225721	C/G	0.000	1	0	1.76E-14
rs73355617	10		106224669	T/G	0.000	1	0	1.76E-14
rs9919362	10		106225815	T/A	0.000	1	0	1.76E-14
rs996083789	10		106224514	T/A	0.002	0	4	1.76E-14
rs73355619	10		106224694	G/A	0.000	1	0	1.76E-14
rs190823253	10		106225017	A/G	0.001	1	1	1.76E-14
rs543177447	10		106225193	C/CT	0.009	7	12	1.76E-14
rs1387868020	10		106225796	A/T	0.005	3	7	1.76E-14
rs1371216199	12	AC010198.2	30819578	T/G	0.000	0	1	7.32E-14

rs1046307021	12	AC010198.2	30820915	G/A	0.000	1	0	7.32E-14
	12	AC010198.2	30819795	T/C	0.000	1	0	7.32E-14
rs1273856379	12	AC010198.2	30820388	C/A	0.000	1	0	7.32E-14
	12		30818926	T/C	0.001	2	1	7.32E-14
	7		121474309	C/T	0.000	1	0	4.62E-13
	7		121474518	G/C	0.000	1	0	4.62E-13
	7		121473754	A/G	0.000	1	0	4.62E-13
	7		121473616	G/A	0.000	1	0	4.62E-13
	7		121474596	C/G	0.000	1	0	4.62E-13
	7		121474761	A/G	0.000	0	1	4.62E-13
rs538891911	7		121472365	C/T	0.003	3	3	4.62E-13
rs183410219	7		121475377	C/T	0.005	5	5	4.62E-13
	7		121475145	T/A	0.001	2	1	4.62E-13
	7		121472485	A/G	0.000	1	0	4.62E-13
	7		121474375	GA/G	0.000	1	0	4.62E-13
	7		121475143	T/TA	0.002	3	1	4.62E-13
	7		121473404	T/C	0.000	1	0	4.62E-13
rs1446787130	7		121472349	C/A	0.003	2	4	4.62E-13
	7		121473151	G/C	0.000	1	0	4.62E-13
	7		121473825	T/G	0.000	1	0	4.62E-13
	7		121472690	A/T	0.000	1	0	4.62E-13
	7		121473071	C/A	0.000	1	0	4.62E-13
	7		121473041	G/A	0.000	1	0	4.62E-13

	7	121472748	A/G	0.000	1	0	4.62E-13
	7	121472571	GT/G	0.001	1	2	4.62E-13
	7	121472786	G/A	0.000	1	0	4.62E-13
rs574734612	7	121473081	C/G	0.001	1	2	4.62E-13
rs371157282	7	121472667	C/T	0.001	1	2	4.62E-13
	7	121472625	A/G	0.000	1	0	4.62E-13
	7	121474944	A/G	0.000	1	0	4.62E-13
rs1461088489	7	121475133	A/G	0.002	1	3	4.62E-13
	7	121475579	A/G	0.000	1	0	4.62E-13
rs1304633099	7	121475575	A/T	0.000	1	0	4.62E-13
	7	121475541	C/T	0.000	1	0	4.62E-13
rs149532000	7	121472003	A/G	0.003	1	6	4.62E-13
	7	121475496	G/T	0.000	1	0	4.62E-13
	7	121475364	T/C	0.000	1	0	4.62E-13
rs1357474141	7	121472370	AAC/A	0.004	5	4	4.62E-13
	7	121473681	A/G	0.000	1	0	4.62E-13
	7	121475098	A/G	0.000	1	0	4.62E-13
	7	121475070	GT/G	0.000	1	0	4.62E-13
	7	121475007	A/AAACT	0.000	1	0	4.62E-13
rs1384916088	10	106227693	ACT/A	0.000	1	0	1.54E-12
rs58841264	10	106227136	G/A	0.000	1	0	1.54E-12
rs1378008779	10	106226865	A/G	0.001	1	1	1.54E-12
rs79743738	10	106226898	C/A	0.000	1	0	1.54E-12

rs12253037	10		106226719	A/G	0.000	1	0	1.54E-12
rs12266228	10		106226697	C/T	0.000	1	0	1.54E-12
rs9919367	10		106226326	T/C	0.000	1	0	1.54E-12
rs896220156	10		106227518	ACT/A	0.004	1	8	1.54E-12
rs9919366	10		106226289	T/C	0.000	1	0	1.54E-12
rs9919365	10		106226128	T/C	0.000	1	0	1.54E-12
rs531719760	10		106227710	TCTC/T	0.005	3	7	1.54E-12
rs9919364	10		106226125	T/C	0.000	1	0	1.54E-12
rs9919497	10		106226116	C/T	0.000	1	0	1.54E-12
	10		106227692	C/T	0.001	0	3	1.54E-12
rs914948342	10		106226226	T/G	0.000	1	0	1.54E-12
rs149446716	10		106227233	C/T	0.000	1	0	1.54E-12
rs181297635	10		106227651	T/C	0.003	4	2	1.54E-12
rs75084758	10		106227332	C/T	0.001	1	2	1.54E-12
	4		135811598	A/C	0.000	1	0	2.32E-12
rs1246745318	4	AC105362.1	135809188	T/C	0.001	1	1	2.32E-12
rs995245842	4	AC105362.1	135808662	G/A	0.001	1	1	2.32E-12
rs369183475	4	AC105362.1	135809425	T/G	0.004	3	6	2.32E-12
	4	AC105362.1	135808311	A/T	0.000	1	0	2.32E-12
rs182924238	4	AC105362.1	135808608	C/T	0.006	5	7	2.32E-12
	4		135808930	T/C	0.000	1	0	2.32E-12
	4		135808466	G/A	0.000	1	0	2.32E-12
	4		135808807	T/G	0.000	1	0	2.32E-12

	4		135808497	C/G	0.000	1	0	2.32E-12
rs375420891	4	AC105362.1	135810873	C/T	0.003	3	3	2.32E-12
	4		135810407	C/A	0.000	1	0	2.32E-12
rs1036573661	4	AC105362.1	135808864	C/A	0.001	1	1	2.32E-12
	4		135808220	T/C	0.000	1	0	2.32E-12
	4		135810000	A/G	0.000	1	0	2.32E-12
	4		135808824	C/T	0.000	1	0	2.32E-12
	4		135809966	G/T	0.000	1	0	2.32E-12
rs375827615	4	AC105362.1	135810546	T/A	0.002	1	3	2.32E-12
rs374515653	4	AC105362.1	135810461	A/T	0.001	2	0	2.32E-12
rs1378926493	4	AC105362.1	135808961	T/C	0.000	1	0	2.32E-12
	4		135809869	C/T	0.000	1	0	2.32E-12
rs1352854037	4	AC105362.1	135809832	G/A	0.001	1	1	2.32E-12
	4		135809474	A/T	0.000	1	0	2.32E-12
	4		135809892	T/C	0.000	1	0	2.32E-12
	4		135808374	G/A	0.000	1	0	2.32E-12
rs1788765	18	ANKRD29	23595596	G/A	0.001	2	0	2.55E-12
rs1788803	18	ANKRD29	23597295	C/T	0.001	2	0	2.55E-12
rs534519893	18	ANKRD29	23594418	C/T	0.005	4	7	2.55E-12
rs184552880	18	ANKRD29	23596913	C/T	0.006	3	10	2.55E-12
rs1004413296	18	ANKRD29	23596455	C/T	0.004	4	4	2.55E-12
rs1411394154	18	ANKRD29	23597546	A/C	0.001	1	1	2.55E-12
rs1436791361	18	ANKRD29	23596624	A/G	0.001	1	1	2.55E-12

rs1652361	18	ANKRD29	23596559	A/G	0.001	2	0	2.55E-12
rs113761973	18	ANKRD29	23594807	C/T	0.001	2	0	2.55E-12
rs1788768	18	ANKRD29	23595461	C/T	0.001	2	0	2.55E-12
rs1788767	18	ANKRD29	23595479	G/T	0.001	2	0	2.55E-12
rs1438284575	18	ANKRD29	23594680	T/A	0.003	3	3	2.55E-12
rs541445238	18	ANKRD29	23596753	C/T	0.002	1	3	2.55E-12
rs543853843	18	ANKRD29	23597442	A/G	0.001	1	2	2.55E-12
rs1788766	18	ANKRD29	23595552	G/A	0.001	2	0	2.55E-12
rs2960581	18	ANKRD29	23596112	C/T	0.001	2	0	2.55E-12
rs976854335	18	ANKRD29	23595436	C/T	0.004	2	7	2.55E-12
rs1652359	18	ANKRD29	23595385	G/A	0.001	2	0	2.55E-12
rs182522197	5	AGXT2	35018407	G/A	0.001	2	1	3.63E-12
	1	GPATCH2	217506372	AG/A	0.001	2	0	1.30E-11
rs1371607670	1	GPATCH2	217504715	G/T	0.000	1	0	1.30E-11
rs192027677	1	GPATCH2	217504543	C/G	0.001	2	1	1.30E-11
rs571499586	1	GPATCH2	217506105	T/C	0.001	2	0	1.30E-11
rs187321686	1	GPATCH2	217506072	C/T	0.006	1	11	1.30E-11
rs1302364041	4	AC105362.1	135807900	C/G	0.001	2	0	2.19E-11
rs1226118749	4	AC105362.1	135806582	C/T	0.001	2	0	2.19E-11
rs189100420	4	AC105362.1	135806106	C/G	0.006	4	9	2.19E-11
	4	AC105362.1	135806753	G/A	0.000	1	0	2.19E-11
	7		121480543	C/T	0.000	1	0	2.37E-11
	7		121480370	A/C	0.000	1	0	2.37E-11

	7	121479978	T/A	0.000	1	0	2.37E-11
rs73427154	7	121479628	A/G	0.001	1	1	2.37E-11
rs1435502580	7	121481474	T/C	0.002	2	2	2.37E-11
rs1416542675	7	121481778	T/C	0.001	1	1	2.37E-11
rs774960171	7	121479876	ACT/A	0.001	0	2	2.37E-11
	7	121481968	G/C	0.000	1	0	2.37E-11
rs78225850	7	121480856	T/C	0.001	1	2	2.37E-11
	7	121478074	C/T	0.000	1	0	2.37E-11
rs1404821314	7	121481108	G/T	0.001	1	1	2.37E-11
rs892020449	7	121480657	A/G	0.002	2	2	2.37E-11
rs1473498433	7	121479011	C/T	0.004	1	7	2.37E-11
rs879405910	7	121479787	G/A	0.001	1	1	2.37E-11
rs533600354	7	121480377	C/T	0.000	0	1	2.37E-11
	7	121480474	T/C	0.000	1	0	2.37E-11
	7	121479737	A/G	0.000	1	0	2.37E-11
	7	121479008	C/T	0.000	1	0	2.37E-11
	7	121478708	T/C	0.000	1	0	2.37E-11
	7	121481134	G/A	0.000	1	0	2.37E-11
	7	121481184	G/A	0.000	1	0	2.37E-11
	7	121478507	T/A	0.000	1	0	2.37E-11
	7	121480611	C/G	0.000	1	0	2.37E-11
	7	121481519	A/T	0.000	1	0	2.37E-11
	7	121481218	T/C	0.000	1	0	2.37E-11

rs73225206	7	121478070	C/T	0.000	1	0	2.37E-11
	7	121480110	C/A	0.000	1	0	2.37E-11
	7	121481291	G/A	0.000	1	0	2.37E-11
	7	121478144	A/G	0.000	1	0	2.37E-11
	7	121481401	A/G	0.000	1	0	2.37E-11
	7	121481512	C/T	0.000	1	0	2.37E-11
rs73717353	7	121481922	T/A	0.002	1	3	2.37E-11
rs142877971	7	121478626	C/A	0.002	1	4	2.37E-11
	7	121478013	C/T	0.000	1	0	2.37E-11
	7	121477558	G/A	0.000	1	0	1.13E-10
rs80315341	7	121476792	C/T	0.004	2	6	1.13E-10
rs578214488	7	121476662	A/T	0.003	3	3	1.13E-10
	7	121477856	G/A	0.000	1	0	1.13E-10
	7	121476016	AT/A	0.000	1	0	1.13E-10
rs1266515091	7	121476440	T/C	0.000	1	0	1.13E-10
	7	121476523	T/C	0.000	1	0	1.13E-10
	7	121476346	CT/C	0.010	3	17	1.13E-10
	7	121476604	T/A	0.000	1	0	1.13E-10
	7	121476781	C/T	0.000	1	0	1.13E-10
rs1007692242	7	121477452	T/C	0.000	1	0	1.13E-10
rs199784006	7	121476252	G/A	0.001	0	2	1.13E-10
	7	121477537	G/A	0.000	1	0	1.13E-10
rs140855148	7	121476661	C/T	0.002	2	2	1.13E-10

rs146647462	7		121477069	G/A	0.004	6	3	1.13E-10
	7	OR2AO1P	144178395	T/C	0.000	1	0	1.91E-10
	7	CTAGE4, OR2AO1P	144178810	A/G	0.000	1	0	1.91E-10
rs1056636222	7	OR2AO1P	144178180	C/A	0.000	1	0	1.91E-10
rs78830825	14		105655778	C/T	0.001	3	0	1.92E-10
rs1319380943	16	C16orf95	87173394	A/C	0.000	1	0	1.99E-10
rs1471706424	16	C16orf95	87171424	A/G	0.002	2	2	1.99E-10
rs1446834578	16	C16orf95	87172215	T/C	0.000	1	0	1.99E-10
rs77820538	16	C16orf95	87170034	C/T	0.001	3	0	1.99E-10
rs571676345	16	C16orf95	87170384	CAA/C	0.001	3	0	1.99E-10
rs191089502	16	C16orf95	87171067	G/A	0.001	1	2	1.99E-10
rs200627555	16	C16orf95	87171966	CTG/C	0.001	2	0	1.99E-10
rs375254150	16	C16orf95	87170326	G/A	0.004	3	6	1.99E-10
rs150010045	16	C16orf95	87172907	T/C	0.004	3	6	1.99E-10
rs933689224	16	C16orf95	87171014	G/A	0.002	4	0	1.99E-10
rs553437092	16	C16orf95	87172056	G/T	0.001	2	1	1.99E-10
rs11647130	16	C16orf95	87170981	T/G	0.002	4	1	1.99E-10
rs563603729	16	C16orf95	87173104	C/A	0.002	3	2	1.99E-10
rs183469474	16	C16orf95	87173089	C/G	0.002	3	2	1.99E-10
rs11640818	16	C16orf95	87171005	C/A	0.002	4	1	1.99E-10
rs1362397428	16	C16orf95	87170829	G/A	0.001	1	1	1.99E-10
rs938499125	16	C16orf95	87170097	C/T	0.000	0	1	1.99E-10

rs1302226558	16	C16orf95	87170466	C/A	0.000	1	0	1.99E-10
rs1432600304	16	C16orf95	87171436	A/G	0.000	1	0	1.99E-10
rs535332768	16	C16orf95	87171738	G/C	0.000	1	0	1.99E-10
rs1016402450	16	C16orf95	87171860	G/C	0.000	1	0	1.99E-10
rs987684197	16	C16orf95	87172034	G/A	0.000	1	0	1.99E-10
rs1162436773	16	C16orf95	87172984	T/C	0.000	1	0	1.99E-10
rs4012343	2	RGPD4	107877863	A/G	0.000	1	0	4.32E-10
rs4012342	2	RGPD4	107877841	A/G	0.001	2	0	4.32E-10
rs746839382	19	C19orf25, APC2	1467456	G/A	0.003	3	3	5.44E-10
rs757776886	19	C19orf25, APC2	1468077	G/A	0.000	1	0	5.44E-10
rs574035458	19	C19orf25, APC2	1469838	C/G	0.002	3	1	5.44E-10
rs758399348	19	C19orf25, APC2	1466670	C/G	0.001	1	1	5.44E-10
rs908761778	19	C19orf25, APC2	1466775	G/A	0.001	1	1	5.44E-10
rs572571000	19	C19orf25, APC2	1467447	G/A	0.001	2	0	5.44E-10
rs527752378	19	C19orf25, APC2	1467102	G/A	0.001	2	0	5.44E-10
rs368193798	19	C19orf25,	1466028	G/A	0.001	1	2	5.44E-10

	19	APC2<br>C19orf25,<br>APC2	1468864	C/T	0.003	4	3	5.44E-10
	7		121482892	C/G	0.000	1	0	6.43E-10
	7		121483369	G/A	0.000	1	0	6.43E-10
	7		121483464	G/A	0.000	1	0	6.43E-10
	7		121482597	AAG/A	0.000	0	1	6.43E-10
rs529012361	7		121482300	T/G	0.002	3	2	6.43E-10
rs563429980	7		121483604	T/C	0.001	0	2	6.43E-10
rs551323577	7		121483771	G/A	0.001	1	1	6.43E-10
rs1405657086	1		60211546	G/A	0.000	1	0	8.49E-10
rs1324963467	1		60210850	A/G	0.001	3	0	8.49E-10
	5		136782505	G/GAC	0.001	3	0	8.64E-10
	5		136782758	T/C	0.001	2	0	8.64E-10
rs143200876	15		99532924	C/T	0.007	7	7	1.00E-09
rs542296951	15		99534625	T/C	0.002	3	2	1.00E-09
rs192883012	15		99532031	T/C	0.001	1	2	1.00E-09
rs1323073113	15		99534543	T/C	0.001	0	3	1.00E-09
rs538034214	15		99532921	TC/T	0.005	9	2	1.00E-09
rs140263304	15		99532160	T/C	0.001	1	2	1.00E-09
rs182366152	15		99535936	T/C	0.001	1	1	1.00E-09
rs753862480	15		99532572	G/A	0.002	2	2	1.00E-09
rs182559181	15		99535257	C/T	0.001	1	1	1.00E-09

rs538239094	15		99534302	T/A	0.000	1	0	1.00E-09
rs1264851546	15		99534389	C/T	0.000	1	0	1.00E-09
rs1227167501	15		99533928	C/T	0.003	3	3	1.00E-09
rs186922193	15		99533056	T/C	0.001	1	1	1.00E-09
rs7183312	15		99532517	C/G	0.004	3	5	1.00E-09
rs543664850	15		99534027	C/T	0.001	1	1	1.00E-09
	15		99530096	A/G	0.000	1	0	1.02E-09
rs569311347	15		99530526	C/A	0.000	1	0	1.02E-09
rs7175687	15		99530234	T/C	0.004	3	6	1.02E-09
rs1233004831	15		99530651	G/T	0.001	1	1	1.02E-09
rs540108096	15		99531042	A/G	0.001	0	2	1.02E-09
rs11864524	16	FAM92B	85109907	G/A	0.001	2	1	1.07E-09
rs13335000	16	FAM92B	85109240	A/G	0.001	2	1	1.07E-09
rs59542409	16	FAM92B	85111051	G/A	0.001	2	1	1.07E-09
rs199599166	16	FAM92B	85108054	C/T	0.002	3	1	1.07E-09
rs111482950	16	FAM92B	85110824	C/G	0.001	2	1	1.07E-09
rs55678605	16	FAM92B	85109370	G/A	0.001	2	1	1.07E-09
rs55711230	16	FAM92B	85109615	A/G	0.001	2	1	1.07E-09
rs55961236	16	FAM92B	85109395	T/C	0.001	2	1	1.07E-09
rs9319455	16	FAM92B	85109955	T/C	0.001	2	1	1.07E-09
rs563454985	16	FAM92B	85110770	T/G	0.003	4	2	1.07E-09
rs9944356	16	FAM92B	85109787	C/T	0.001	2	1	1.07E-09
rs9944358	16	FAM92B	85109827	G/T	0.001	2	1	1.07E-09

rs765272850	16	FAM92B	85110461	C/G	0.001	1	1	1.07E-09
rs28760437	16	FAM92B	85111495	C/T	0.001	2	1	1.07E-09
rs9319456	16	FAM92B	85109958	G/A	0.001	2	1	1.07E-09
	16	FAM92B	85109250	AT/A	0.002	3	2	1.07E-09
rs139162213	16	FAM92B	85108279	C/G	0.004	3	5	1.07E-09
rs911620841	16	FAM92B	85111773	G/A	0.002	1	4	1.07E-09
rs148579640	16	FAM92B	85108490	G/A	0.005	5	5	1.07E-09
rs375852815	16	FAM92B	85111400	C/T	0.008	10	6	1.07E-09
rs375723278	16	FAM92B	85108014	G/A	0.001	2	0	1.07E-09
rs1290129195	16	FAM92B	85110748	A/C	0.001	0	2	1.07E-09
rs1202653382	16	FAM92B	85111028	A/C	0.001	2	0	1.07E-09
rs953679194	16	FAM92B	85110098	G/C	0.001	1	2	1.07E-09
rs554595302	16	FAM92B	85109486	G/A	0.004	1	8	1.07E-09
rs56161425	16	FAM92B	85109239	C/T	0.001	2	1	1.07E-09
	16	FAM92B	85111415	G/A	0.001	0	3	1.07E-09
rs17200833	16	FAM92B	85108055	G/A	0.001	2	1	1.07E-09
rs72807613	16	FAM92B	85108366	T/C	0.001	2	1	1.07E-09
rs113907412	16	FAM92B	85108670	C/G	0.001	2	1	1.07E-09
rs9931416	16	FAM92B	85108816	A/G	0.001	2	1	1.07E-09
rs55837879	16	FAM92B	85109028	C/T	0.001	2	1	1.07E-09
rs56102116	16	FAM92B	85109061	T/C	0.001	2	1	1.07E-09
rs531362239	16	FAM92B	85111651	C/T	0.001	2	1	1.07E-09
rs753091695	16	FAM92B	85110392	G/A	0.004	3	5	1.07E-09

rs56231750	16	FAM92B	85109148	T/C	0.001	2	1	1.07E-09
rs1270188757	13	SUPT20H	37062435	T/C	0.001	2	1	2.13E-09
rs372613721	13	SUPT20H	37064009	T/C	0.003	3	3	2.13E-09
	13		37063913	T/C	0.001	1	1	2.13E-09
rs1371784881	13	SUPT20H	37064091	A/G	0.001	2	1	2.13E-09
rs201615420	13	SUPT20H	37064498	A/G	0.001	1	1	2.13E-09
rs59899061	13	SUPT20H	37062302	C/T	0.003	0	7	2.13E-09
rs368631110	13	SUPT20H	37063131	T/G	0.003	2	5	2.13E-09
	13		37062189	C/T	0.001	1	2	2.13E-09
	13		37062603	A/G	0.001	1	2	2.13E-09
	13		37062826	A/G	0.001	1	2	2.13E-09
rs1296292334	13		37064983	GTGTGTGTGTGTGTA/G	0.001	2	0	2.13E-09
rs2801796	13	SUPT20H	37063552	C/A	0.001	0	2	2.13E-09
rs185784371	13	SUPT20H	37062308	T/C	0.001	0	2	2.13E-09
rs1347974842	13	SUPT20H	37063238	G/C	0.001	1	2	2.13E-09
rs184629730	13	SUPT20H	37064640	T/C	0.000	1	0	2.13E-09
rs189839938	13		37065364	G/A	0.001	1	2	2.13E-09
	13		37065434	C/T	0.001	1	2	2.13E-09
rs535403515	13	SUPT20H	37062171	A/G	0.000	1	0	2.13E-09
rs146753277	13	SUPT20H	37064169	T/C	0.007	10	4	2.13E-09
rs1031567515	13		37066065	G/A	0.000	0	1	2.16E-09
rs1267585733	13		37066949	G/A	0.000	1	0	2.16E-09
rs544581365	13		37067740	G/A	0.000	1	0	2.16E-09

rs926878466	13		37067824	A/G	0.000	0	1	2.16E-09
rs1359426229	13		37067584	T/C	0.001	2	1	2.16E-09
rs1341530945	13		37067649	G/GT	0.003	2	5	2.16E-09
rs745873863	13		37067045	TATGTATGTATGTATG/T	0.004	1	7	2.16E-09
rs1022808628	13		37066502	T/G	0.001	0	2	2.16E-09
	13		37067739	C/T	0.001	1	2	2.16E-09
	13		37067500	G/A	0.001	1	2	2.16E-09
rs1185571895	13		37066320	G/C	0.001	3	0	2.16E-09
rs374547208	13		37066269	G/C	0.004	5	3	2.16E-09
	13		37067677	C/G	0.001	1	1	2.16E-09
rs1217923798	13		37067043	TG/T	0.004	1	7	2.16E-09
	13		37066849	C/CT	0.001	1	1	2.16E-09
	13		37066994	C/T	0.001	1	2	2.16E-09
rs190207292	13		37066351	A/T	0.001	1	2	2.16E-09
rs1035006069	13		37066573	C/G	0.002	2	3	2.16E-09
rs75892233	13	SUPT20H	37036330	T/G	0.002	3	1	2.19E-09
rs1376676427	13	SUPT20H	37036377	T/G	0.001	1	2	2.19E-09
rs1288225696	13	SUPT20H	37038684	T/C	0.001	0	3	2.19E-09
	13		37037241	T/G	0.001	1	2	2.19E-09
rs548390251	13	SUPT20H	37038083	T/A	0.001	1	2	2.19E-09
	13		37038470	C/T	0.001	1	2	2.19E-09
	13		37039186	C/T	0.001	1	2	2.19E-09
rs150439276	13	SUPT20H	37037074	A/G	0.007	10	4	2.19E-09

	13	SUPT20H	37037038	T/TA	0.003	3	4	2.19E-09
rs1461517132	13	SUPT20H	37039361	G/A	0.002	1	4	2.19E-09
rs1357277555	13	SUPT20H	37036298	T/C	0.001	0	3	2.19E-09
	13		37036796	T/C	0.001	1	2	2.19E-09
	13		37037832	G/A	0.001	1	2	2.19E-09
	13		37036430	C/T	0.001	1	2	2.19E-09
rs3781276	10	PDE6C	93625853	T/A	0.008	6	11	2.20E-09
rs185913538	10	PDE6C	93622127	C/A	0.001	2	0	2.20E-09
rs539470997	10	PDE6C	93625971	G/A	0.000	1	0	2.20E-09
rs140756004	12		10490591	G/A	0.001	2	0	2.53E-09
rs138464950	12		10491751	C/T	0.001	1	2	2.53E-09
rs372587641	12		10493588	A/T	0.005	2	8	2.53E-09
rs531158543	12		10493640	T/A	0.001	0	2	2.53E-09
rs1275175704	12		10493550	GT/G	0.001	2	0	2.53E-09
rs113260792	12		10491008	C/T	0.001	2	0	2.53E-09
rs541620279	12		10492869	T/C	0.001	1	1	2.53E-09
rs183543816	12		10490003	G/T	0.006	7	5	2.53E-09
rs1392128324	12		10491586	TTAATC/T	0.000	1	0	2.53E-09
rs1475570291	12		10492576	A/G	0.002	1	3	2.53E-09
rs201405639	12		10490251	T/A	0.000	0	1	2.53E-09
rs890517176	12		10489797	T/C	0.004	1	8	2.60E-09
rs541677115	12		10489358	TA/T	0.003	1	6	2.60E-09
rs77904948	12		10489582	G/A	0.002	1	3	2.60E-09

rs191365206	12		10489993	C/T	0.002	0	4	2.60E-09
rs1252074479	12		10488616	A/G	0.001	0	2	2.60E-09
rs182666816	12		10489930	T/C	0.001	2	1	2.60E-09
rs1258043800	12		10489523	A/G	0.001	0	3	2.60E-09
rs1478313462	12		10489379	T/A	0.001	1	1	2.60E-09
	12		10488373	T/C	0.002	2	3	2.60E-09
rs1200964206	12		10488332	T/C	0.001	1	1	2.60E-09
rs548790422	13	SUPT20H	37034951	T/A	0.001	2	0	4.11E-09
	13	SUPT20H	37034399	T/A	0.001	1	1	4.11E-09
rs1487544133	13	SUPT20H	37035933	CA/C	0.000	1	0	4.11E-09
rs562712453	13	SUPT20H	37035888	C/T	0.008	3	14	4.11E-09
rs142078227	13	SUPT20H	37034856	T/C	0.000	0	1	4.11E-09
	13	SUPT20H	37035524	G/GT	0.002	3	2	4.11E-09
	13		37034529	A/G	0.001	1	2	4.11E-09
	13		37035588	T/C	0.001	1	2	4.11E-09
rs1309139294	2	4-Mar	216327978	AAAAG/A	0.001	2	0	4.48E-09
rs376556970	2	4-Mar	216327443	A/G	0.000	1	0	4.48E-09
	2	4-Mar	216327148	AT/A	0.009	6	12	4.48E-09
	1	RALGPS2	178768295	T/C	0.000	0	1	5.04E-09
rs115983069	1	RALGPS2	178768543	A/G	0.008	5	11	5.04E-09
rs146993806	1	RALGPS2	178771531	TCA/T	0.007	8	7	5.04E-09
rs940361146	1	RALGPS2	178771128	C/T	0.001	1	2	5.04E-09
rs148751159	1	RALGPS2	178770651	A/G	0.008	5	11	5.04E-09

rs146929160	1	RALGPS2	178768848	T/C	0.002	1	3	5.04E-09
	1	RALGPS2	178770447	C/T	0.001	0	3	5.04E-09
rs556007526	1	RALGPS2	178770315	T/C	0.009	6	12	5.04E-09
rs140767296	1	RALGPS2	178769099	A/G	0.000	0	1	5.04E-09
rs1265735251	1	RALGPS2	178769082	G/A	0.001	1	2	5.04E-09
rs1311275848	1	RALGPS2	178768095	A/C	0.002	2	2	5.04E-09
rs112972340	1	RALGPS2	178770150	G/A	0.008	5	11	5.04E-09
rs114338724	1	RALGPS2	178770194	T/C	0.008	5	11	5.04E-09
rs561654540	1	RALGPS2	178768428	G/A	0.000	1	0	5.04E-09
rs1394967875	1	RALGPS2	178769431	G/A	0.001	2	1	5.04E-09
rs1415093332	1	RALGPS2	178771024	A/G	0.001	1	1	5.04E-09
	1	RALGPS2	178771574	A/AT	0.008	5	12	5.04E-09
rs1316270754	1	RALGPS2	178770437	T/A	0.001	2	1	5.04E-09
rs368551181	1	RALGPS2	178769669	G/A	0.007	4	10	5.04E-09
rs1047138481	1	RALGPS2	178773589	A/G	0.000	0	1	5.15E-09
rs74681102	1	RALGPS2	178773537	A/T	0.008	5	11	5.15E-09
rs79005101	1	RALGPS2	178773432	A/G	0.008	5	11	5.15E-09
rs185618684	1	RALGPS2	178772029	C/T	0.004	6	3	5.15E-09
	1	RALGPS2	178773449	T/A	0.000	0	1	5.15E-09
rs548011370	1	RALGPS2	178772036	T/C	0.004	5	4	5.15E-09
rs551833386	1	RALGPS2	178773286	A/G	0.000	0	1	5.15E-09
rs11811840	1	RALGPS2	178772992	G/A	0.002	1	3	5.15E-09
rs1186903271	19	OR7E25P,	9213612	C/T	0.001	1	1	5.32E-09

		OR7D4						
rs185770505	19	OR7D4	9215518	T/C	0.005	7	4	5.32E-09
rs1295402112	19	OR7E25P, OR7D4	9213784	A/G	0.001	1	1	5.32E-09
rs559806910	19	OR7E25P, OR7D4	9213245	A/C	0.000	1	0	5.32E-09
rs192169587	19	OR7D4, OR7E25P	9212477	A/G	0.001	1	2	5.32E-09
rs746009560	19	OR7D4, OR7E25P	9214314	G/A	0.001	1	1	5.32E-09
rs5020280	19	OR7D4, OR7E25P	9214587	C/T	0.010	8	12	5.32E-09
rs571345794	19	OR7D4, OR7E25P	9212815	C/T	0.004	3	6	5.32E-09
rs138543083	19	OR7E25P, OR7D4	9213236	C/G	0.001	1	1	5.32E-09
rs368164789	19	OR7D4, OR7E25P	9212142	C/T	0.005	1	10	5.32E-09
rs1011866816	19	OR7D4	9217758	C/T	0.001	1	2	5.57E-09
rs549886736	19	OR7D4	9217167	C/T	0.003	4	2	5.57E-09
rs187498402	19	OR7D4	9217129	G/C	0.003	2	4	5.57E-09
rs191891240	19	OR7D4	9216833	C/T	0.006	2	11	5.57E-09
	19	OR7D4	9217572	T/C	0.000	1	0	5.57E-09

rs182565540	19	OR7D4	9217899	A/G	0.001	2	0	5.57E-09
rs570729575	1		178629127	T/C	0.001	1	1	6.34E-09
rs189806094	1		178631974	C/T	0.002	1	3	6.34E-09
rs1422105212	1		178629005	AGCCAT/A	0.001	1	2	6.34E-09
rs1380369235	1		178631071	T/C	0.001	1	1	6.34E-09
rs1359254694	1		178631761	CCG/C	0.007	7	7	6.34E-09
rs1323965623	1		178628474	G/T	0.001	0	2	6.34E-09
rs570648630	1		178628333	C/A	0.003	2	5	6.34E-09
rs559954634	1		178632863	C/T	0.003	5	2	6.36E-09
rs1998543	1		178632952	C/T	0.001	1	2	6.36E-09
rs571949288	1		178632729	C/T	0.000	1	0	6.36E-09
rs117999754	15	PLA2G4D	42092094	A/C	0.000	1	0	7.14E-09
rs74554336	15	PLA2G4D	42092323	G/A	0.000	1	0	7.14E-09
rs1427992315	15	PLA2G4D	42093895	G/T	0.001	2	0	7.14E-09
rs7162019	15	PLA2G4D	42093419	G/A	0.000	1	0	7.14E-09
rs116953180	15	PLA2G4D	42092092	T/C	0.000	1	0	7.14E-09
rs144925289	15	PLA2G4D	42092129	C/T	0.003	2	5	7.14E-09
rs17748132	15	PLA2G4D	42093804	C/T	0.000	1	0	7.14E-09
rs1030848045	15	PLA2G4D	42093825	C/T	0.000	0	1	7.14E-09
rs78171718	15	PLA2G4D	42094144	C/T	0.000	1	0	7.14E-09
rs76779748	15	PLA2G4D	42094187	G/A	0.000	1	0	7.14E-09
rs575744349	15	PLA2G4D	42092981	G/T	0.000	1	0	7.14E-09
	15	PLA2G4D	42092803	T/G	0.001	2	0	7.14E-09

rs561017822	15	PLA2G4D	42093462	C/T	0.002	0	4	7.14E-09
rs560714345	15	PLA2G4D	42093533	C/T	0.001	1	2	7.14E-09
rs112407457	15	PLA2G4D	42092108	C/T	0.001	1	1	7.14E-09
rs75615772	15	PLA2G4D	42094282	C/T	0.000	1	0	7.14E-09
rs77356331	15	PLA2G4D	42095074	C/T	0.000	1	0	7.14E-09
rs8029713	15	PLA2G4D	42094920	A/G	0.000	1	0	7.14E-09
rs11316676	15	PLA2G4D	42094871	AG/A	0.000	1	0	7.14E-09
rs1254084508	15	PLA2G4D	42095821	A/G	0.001	1	1	7.14E-09
rs75734921	15	PLA2G4D	42094432	G/T	0.000	1	0	7.14E-09
rs187896855	15	PLA2G4D	42095978	G/A	0.006	8	4	7.14E-09
rs1034067834	15	PLA2G4D	42094153	T/G	0.000	0	1	7.14E-09
rs1474004987	14	AL122127.1	105784525	C/T	0.001	1	1	7.19E-09
rs1455059355	14	AL122127.1	105784418	A/G	0.000	1	0	7.19E-09
	14	AL122127.1	105784964	A/G	0.000	1	0	7.19E-09
rs369377100	14	AL122127.1	105785054	G/T	0.005	4	7	7.19E-09
	14	AL122127.1	105785033	G/GT	0.002	4	0	7.19E-09
rs887252312	14	AL122127.1	105785309	G/A	0.000	1	0	7.19E-09
rs1269199687	7		121470045	C/T	0.001	1	1	1.05E-08
rs574165653	7		121471398	T/C	0.000	1	0	1.05E-08
rs1159745552	7		121470874	C/T	0.001	2	0	1.05E-08
	7		121471872	G/A	0.000	1	0	1.05E-08
rs547659893	7		121470474	A/G	0.002	3	2	1.05E-08
rs545053079	7		121471878	C/T	0.005	2	9	1.05E-08

rs191056852	11	AC090592.1	25749637	G/A	0.006	2	11	1.07E-08
rs1306710671	11	AC090592.1	25750721	T/C	0.001	2	1	1.07E-08
rs542014538	11	AC090592.1	25751332	C/T	0.001	3	0	1.07E-08
rs374832061	11	AC090592.1	25751625	T/A	0.005	3	8	1.07E-08
rs1028912802	11	AC090592.1	25748511	C/T	0.005	3	8	1.07E-08
rs1389910862	11	AC090592.1	25751216	G/C	0.002	3	1	1.07E-08
rs192872561	11	AC090592.1	25749758	C/T	0.001	2	0	1.07E-08
rs1476340106	11	AC090592.1	25751960	A/G	0.001	2	0	1.07E-08
rs897253804	9	AL162726.3	82529593	C/A	0.000	1	0	1.14E-08
rs745657928	9	AL162726.3	82527931	T/C	0.000	1	0	1.14E-08
	9	AL162726.3	82526995	A/G	0.000	0	1	1.14E-08
rs1289000741	9	AL162726.3	82527217	T/G	0.001	1	1	1.14E-08
rs183093348	9	AL162726.3	82529216	T/G	0.001	1	1	1.14E-08
rs75958836	9	AL162726.3	82527191	C/T	0.008	6	11	1.14E-08
rs75279437	9	AL162726.3	82527125	G/A	0.002	2	2	1.14E-08
rs139673820	9	AL162726.3	82528142	C/T	0.005	8	2	1.14E-08
rs1010061421	9	AL162726.3	82529963	A/G	0.003	3	4	1.14E-08
rs62576848	9	AL162726.3	82529762	C/T	0.001	2	0	1.14E-08
rs146156181	9	AL162726.3	82526212	G/T	0.001	2	0	1.14E-08
rs544161239	16	FAM92B	85107351	G/A	0.001	2	0	2.05E-08
rs574388332	16	FAM92B	85107003	A/G	0.000	0	1	2.05E-08
	16	FAM92B	85106549	G/C	0.000	1	0	2.05E-08
rs372242153	16	FAM92B	85107335	G/A	0.007	5	9	2.05E-08

rs1490164931	16	FAM92B	85106418	G/T	0.000	1	0	2.05E-08
rs949322733	16	FAM92B	85107551	C/T	0.002	1	3	2.05E-08
rs1461975755	16	FAM92B	85107517	T/C	0.001	2	1	2.05E-08
rs67587184	16	FAM92B	85107950	G/A	0.001	2	1	2.05E-08
rs17200798	16	FAM92B	85107686	T/C	0.002	2	2	2.05E-08
rs548733847	16	FAM92B	85107582	A/G	0.001	1	1	2.05E-08
rs181228667	16	FAM92B	85106513	G/A	0.002	4	1	2.05E-08
rs189692646	16	FAM92B	85107601	G/A	0.008	4	12	2.05E-08
rs914907926	16	FAM92B	85106759	G/A	0.003	3	3	2.05E-08
rs1367358399	16	FAM92B	85107090	G/A	0.001	0	3	2.05E-08
rs77381973	16	FAM92B	85106907	G/C	0.002	1	4	2.05E-08
rs979999112	18		39026909	T/C	0.000	0	1	2.23E-08
rs139503168	18		39026321	T/C	0.003	4	3	2.23E-08
rs16971493	18		39027983	A/C	0.000	1	0	2.23E-08
rs184539612	18		39025361	C/A	0.002	1	4	2.23E-08
rs16971490	18		39027943	C/T	0.000	1	0	2.23E-08
	18		39027785	T/C	0.000	0	1	2.23E-08
rs77675394	18		39027680	A/C	0.000	1	0	2.23E-08
rs57012644	18		39027584	G/A	0.000	1	0	2.23E-08
rs1485225053	18		39027007	A/T	0.000	1	0	2.23E-08
rs16971486	18		39026515	C/T	0.000	1	0	2.23E-08
rs1568095107	18		39025042	T/C	0.001	2	0	2.23E-08
rs1447484432	18		39026967	T/C	0.001	1	1	2.23E-08

rs149517677	18		39024742	T/G	0.001	1	2	2.23E-08
rs950806939	18		39027469	G/A	0.001	1	1	2.23E-08
rs60196408	18		39026403	C/T	0.000	1	0	2.23E-08
rs569346006	18		39024360	C/G	0.001	1	1	2.23E-08
	18		39024436	C/T	0.000	0	1	2.23E-08
rs374743667	18		39024475	C/G	0.000	0	1	2.23E-08
rs375422213	18		39025897	G/T	0.001	1	2	2.23E-08
rs7242940	18		39025216	C/T	0.000	1	0	2.23E-08
rs7242951	18		39025273	A/G	0.000	1	0	2.23E-08
rs16971476	18		39025549	C/T	0.000	1	0	2.23E-08
rs1336729896	18		39026823	T/A	0.001	2	1	2.23E-08
rs144030951	18		39025679	GC/G	0.000	1	0	2.23E-08
rs7242644	18		39025108	A/G	0.000	1	0	2.23E-08
rs191475088	8	LINC02055	136743749	T/C	0.003	5	1	2.51E-08
rs1461302739	8	LINC02055	136742152	C/T	0.002	4	0	2.51E-08
rs140417700	8	LINC02055	136743628	G/A	0.006	4	8	2.51E-08
rs1348094581	8	LINC02055	136744734	T/G	0.001	2	0	2.51E-08
rs143095981	8	LINC02055	136744130	C/T	0.001	2	1	2.51E-08
rs148031375	20		11876652	C/G	0.003	2	4	3.03E-08
rs1230314920	20		11876767	C/A	0.001	1	2	3.03E-08
rs192597288	20		11878556	C/T	0.004	7	2	3.03E-08
rs75755442	20		11878042	G/A	0.007	7	8	3.03E-08
rs571768374	20		11879761	T/C	0.000	1	0	3.03E-08

rs1344344694	20		11878021	ATAGT/A	0.001	1	1	3.03E-08
rs185777070	20		11877405	C/G	0.001	0	2	3.03E-08
rs1268672503	20		11878740	T/A	0.001	0	2	3.03E-08
rs776925152	20		11876046	A/G	0.000	0	1	3.03E-08
rs59282571	20		11878543	G/C	0.000	1	0	3.03E-08
rs200909907	20		11877823	C/T	0.001	1	1	3.03E-08
rs529009910	20		11878639	T/C	0.003	1	5	3.03E-08
	1	NFIA	61147140	C/A	0.001	2	0	3.15E-08
rs1037624099	1	NFIA	61147121	A/G	0.006	7	6	3.15E-08
rs542315710	1	NFIA	61147410	C/T	0.001	0	2	3.15E-08
rs186763678	1	NFIA	61147493	G/T	0.004	1	8	3.15E-08
rs187715568	1	NFIA	61149509	A/G	0.001	1	2	3.15E-08
rs559013047	1	NFIA	61146320	T/TAGGATATAGACATC	0.007	7	7	3.15E-08
	1	NFIA	61147652	G/GT	0.001	1	1	3.15E-08
rs529975259	1	NFIA	61148186	G/A	0.000	1	0	3.15E-08
rs1272906087	1	NFIA	61147249	C/T	0.001	1	1	3.15E-08
	1	NFIA	61148105	A/G	0.000	0	1	3.15E-08
rs138122576	1	NFIA	61146345	G/T	0.004	2	6	3.15E-08
rs1320741915	1	NFIA	61147611	G/A	0.000	1	0	3.15E-08
rs939589014	1	NFIA	61147419	A/G	0.000	1	0	3.15E-08
	1	NFIA	61148981	T/C	0.000	0	1	3.15E-08
rs147403465	1	NFIA	61147592	G/A	0.002	1	3	3.15E-08
rs559601625	1	NFIA	61146098	T/G	0.000	1	0	3.15E-08

rs1004248127	1	NFIA	61146440	A/G	0.002	2	2	3.15E-08
rs180833632	1	NFIA	61147013	C/T	0.000	0	1	3.15E-08
rs1213688759	1	NFIA	61146525	A/C	0.001	2	1	3.15E-08
rs1055712320	1	NFIA	61145933	A/G	0.001	2	1	3.18E-08
rs141022609	1	NFIA	61144749	C/T	0.000	0	1	3.18E-08
rs148897458	8	C8orf34-AS1	68311962	C/A	0.002	0	5	3.40E-08
rs1048223509	8	C8orf34-AS1	68313148	G/T	0.002	2	2	3.40E-08
rs184310318	8	C8orf34-AS1	68311354	C/T	0.003	3	3	3.40E-08
rs74369862	8	C8orf34-AS1	68310517	A/C	0.001	0	3	3.40E-08
rs74967966	8	C8orf34-AS1	68310539	T/A	0.001	0	3	3.40E-08
rs1436509432	8	C8orf34-AS1	68311755	C/T	0.001	2	1	3.40E-08
rs543716184	8	C8orf34-AS1	68311321	C/CT	0.005	2	8	3.40E-08
	8	C8orf34-AS1	68310993	C/CCTTCCTTCCTTCCTTCCTTTCTAT	0.000	0	1	3.40E-08
rs146660093	8	C8orf34-AS1	68310330	C/T	0.007	7	7	3.40E-08
rs527720127	8	C8orf34-AS1	68313253	T/C	0.001	0	2	3.40E-08
rs73262970	8	C8orf34-AS1	68312301	C/T	0.001	0	2	3.40E-08
rs190435824	8	C8orf34-AS1	68312396	G/A	0.002	1	3	3.40E-08
rs1017354638	8	C8orf34-AS1	68313999	T/C	0.001	1	1	3.40E-08
rs111388404	8	C8orf34-AS1	68310882	C/T	0.001	1	1	3.40E-08
rs552491376	8	C8orf34-AS1	68310051	T/G	0.004	5	3	3.40E-08
rs1229854620	8	C8orf34-AS1	68310982	TTC/T	0.001	1	2	3.40E-08
rs372194511	8	C8orf34-AS1	68313947	A/T	0.003	1	5	3.40E-08
rs1232453654	8	C8orf34-AS1	68311611	C/T	0.000	1	0	3.40E-08

rs146399596	8	C8orf34-AS1	68313577	T/G	0.000	0	1	3.40E-08
rs1278707825	13		85338562	C/T	0.001	1	1	3.57E-08
rs1242148640	13		85341753	GA/G	0.004	1	7	3.57E-08
	13		85340105	TTTA/T	0.002	0	5	3.57E-08
	13		85340778	G/A	0.003	6	1	3.57E-08
	13		85340782	G/A	0.006	11	1	3.57E-08
rs947550584	13		85338565	T/A	0.001	2	1	3.57E-08
rs182333891	13		85338226	G/A	0.000	1	0	3.57E-08
rs1212231845	13		85338575	G/A	0.002	1	4	3.57E-08
rs80011615	13		85338724	G/A	0.004	1	8	3.57E-08
	13		85340121	T/TA	0.001	0	2	3.57E-08
rs537061475	13		85339507	C/T	0.001	1	1	3.57E-08
rs115851984	13		85339596	T/C	0.004	1	8	3.57E-08
rs114193933	13		85341653	T/G	0.004	1	8	3.57E-08
rs77229876	13		85341950	C/T	0.004	1	8	3.57E-08
rs1398548051	13		85339060	C/T	0.001	1	1	3.57E-08
rs866277015	13		85341951	G/A	0.001	1	2	3.57E-08
