# Supplementary material for: Whole-genome sequencing reveals rare variants associated with gout in Taiwanese males
Source: Front Genet. 2024 Sep 25;15:1423714. doi: 10.3389/fgene.2024.1423714 (PMC11462091; doi:10.3389/fgene.2024.1423714)
Supplement: Supplementary file 3 [file DataSheet1.PDF]

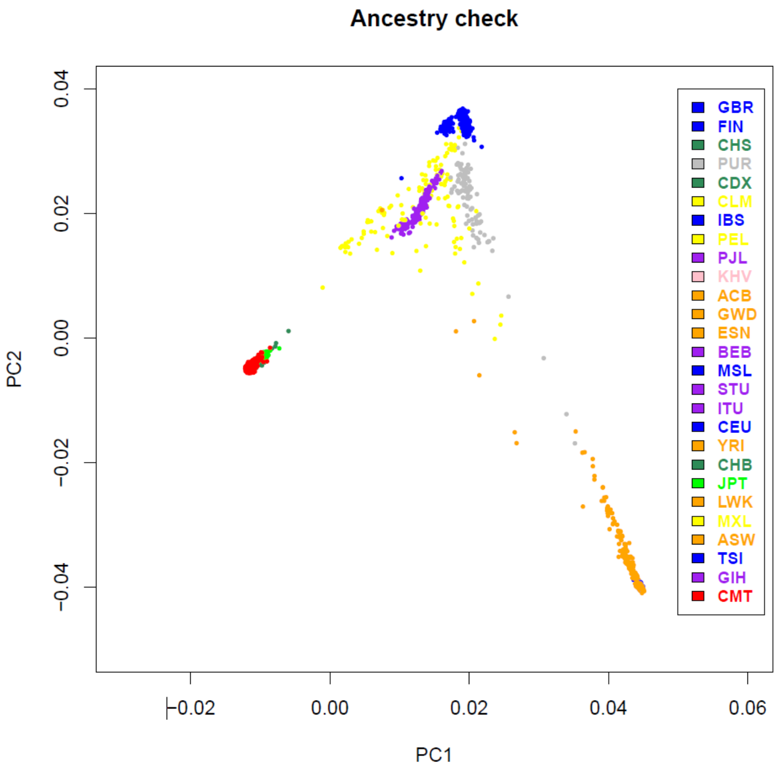

Figure S1A. 1280 subjects with ancestry information of 1000 genome were combined with our samples (CMUH_TWB). After LD pruning, SNPs with genotyping call rate >95% and MAF >1% were plotted against PC1 and PC2, where CMT (red dots) denote CMUH_TWB.

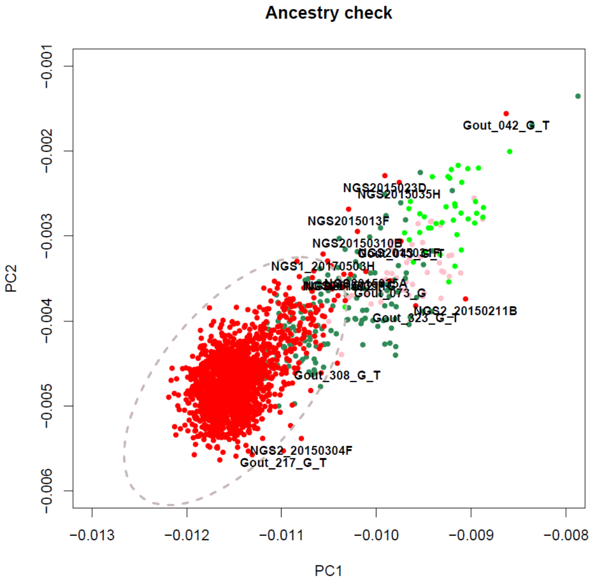

Figure S1B. We zoomed into our samples, where the dotted elliptical denotes the 99.9% confidence bound (CB). We excluded samples outside the CB. Thus, all samples analyzed subsequently share the same genetic background.

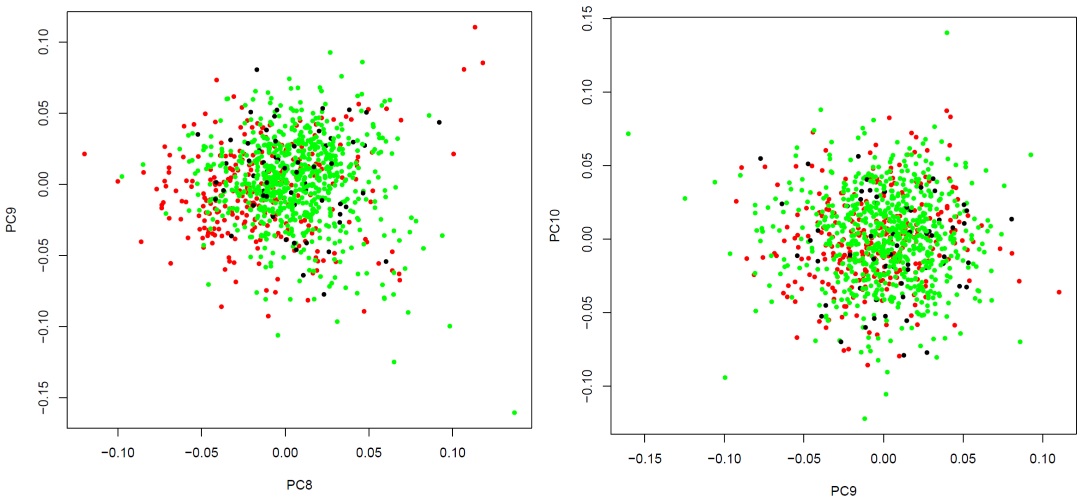

Figure S2. The PCA plots show that when plotted against PC9 and PC10, the subjects of the CMUH and TWB are well mixed, where the red, green and black dots denote cases (gout patients) from CMUH, controls and cases from TWB, respectively.
